# NS5A inhibitors unmask differences in functional replicase complex half-life between different hepatitis C virus strains

**DOI:** 10.1371/journal.ppat.1006343

**Published:** 2017-06-08

**Authors:** Tiffany Benzine, Ryan Brandt, William C. Lovell, Daisuke Yamane, Petra Neddermann, Raffaele De Francesco, Stanley M. Lemon, Alan S. Perelson, Ruian Ke, David R. McGivern

**Affiliations:** 1 Lineberger Comprehensive Cancer Center, University of North Carolina at Chapel Hill, Chapel Hill, North Carolina, United States of America; 2 Division of Infectious Diseases, Department of Medicine, University of North Carolina at Chapel Hill, Chapel Hill, North Carolina, United States of America; 3 Department of Mathematics, North Carolina State University, Raleigh, North Carolina, United States of America; 4 INGM -Istituto Nazionale di Genetica Molecolare "Romeo ed Enrica Invernizzi", Milan, Italy; 5 Theoretical Biology and Biophysics, Los Alamos National Laboratory, Los Alamos, New Mexico, United States of America; The University of Chicago, UNITED STATES

## Abstract

Hepatitis C virus (HCV) RNA is synthesized by the replicase complex (RC), a macromolecular assembly composed of viral non-structural proteins and cellular co-factors. Inhibitors of the HCV NS5A protein block formation of new RCs but do not affect RNA synthesis by pre-formed RCs. Without new RC formation, existing RCs turn over and are eventually lost from the cell. We aimed to use NS5A inhibitors to estimate the half-life of the functional RC of HCV. We compared different cell culture-infectious strains of HCV that may be grouped based on their sensitivity to lipid peroxidation: robustly replicating, lipid peroxidation resistant (LPO^R^) viruses (e.g. JFH-1 or H77D) and more slowly replicating, lipid peroxidation sensitive (LPO^S^) viruses (e.g. H77S.3 and N.2). In luciferase assays, LPO^S^ HCV strains declined under NS5A inhibitor therapy with much slower kinetics compared to LPO^R^ HCV strains. This difference in rate of decline was not observed for inhibitors of the NS5B RNA-dependent RNA polymerase suggesting that the difference was not simply a consequence of differences in RNA stability. In further analyses, we compared two isoclonal HCV variants: the LPO^S^ H77S.3 and the LPO^R^ H77D that differ only by 12 amino acids. Differences in rate of decline between H77S.3 and H77D following NS5A inhibitor addition were not due to amino acid sequences in NS5A but rather due to a combination of amino acid differences in the non-structural proteins that make up the HCV RC. Mathematical modeling of intracellular HCV RNA dynamics suggested that differences in RC stability (half-lives of 3.5 and 9.9 hours, for H77D and H77S.3, respectively) are responsible for the different kinetics of antiviral suppression between LPO^S^ and LPO^R^ viruses. In nascent RNA capture assays, the rate of RNA synthesis decline following NS5A inhibitor addition was significantly faster for H77D compared to H77S.3 indicating different half-lives of functional RCs.

## Author summary

Inhibitors targeting the HCV NS5A protein are a key component of highly effective interferon-free combination therapies for chronic hepatitis C. Despite their high potency against HCV, the precise details of their mode of action are poorly understood. They are known to block assembly and release of virus particles from infected hepatocytes, resulting in a rapid drop in viral RNA in the blood. Additionally they block formation of intracellular membrane structures that are the site of viral RNA synthesis in infected hepatocytes. By preventing membrane remodeling, NS5A inhibitors effectively block formation of new RCs within the cell. Following addition of NS5A inhibitors to infected cell cultures, the kinetics of antiviral suppression were found to vary between different HCV strains, independent of specific differences in NS5A sequence. Using an integrated experimental and mathematical modeling approach, we provide evidence that the rate of decline of viral RNA abundance in infected cells treated with NS5A inhibitors is determined by the stability or half-life of the functional HCV RC.

## Introduction

Direct-acting antivirals (DAAs) targeting the hepatitis C virus (HCV) include specific inhibitors of the NS3/4A protease/helicase, the NS5B RNA-dependent RNA polymerase and the NS5A protein. Combination therapies with two or more DAAs can result in a sustained virological response (SVR) in most infected persons and have revolutionized treatment of chronic hepatitis C in the USA and other developed countries.

Inhibitors targeting NS5A are a key component of antiviral regimens currently used in the clinic. These include ledipasvir, daclatasvir, ombitasvir, elbasvir and velpatasvir. Next generation NS5A inhibitors in clinical development include, ruzasvir, pibrentasvir and odalasvir. NS5A inhibitors were originally identified by screening libraries of compounds for antiviral activity in cell-based screening assays [1]. NS5A was identified as the target of this class of drug by streptavidin pulldown of a biotinylated inhibitor from lysates of HCV-infected cells and also by sequence analysis of drug-resistant replicons. Initially, the mode of action of this class of drug was unclear since NS5A has no known enzymatic activity and its structure is only partly characterized. Furthermore, NS5A is a multifunctional protein that participates in several processes in the viral life cycle. Recent studies have shed some light on the mode of action of NS5A inhibitors but molecular mechanisms remain incompletely characterized.

Studies of NS5A inhibitors in vitro [2] and in vivo [3] suggest a dual mode of action with inhibition of both viral RNA synthesis and virion assembly. The molecular mechanisms underlying NS5A inhibitor blockade of RNA synthesis have been studied in greatest detail but much remains uncharacterized. HCV RNA genomes are synthesized by multi-protein replicase complexes (RCs) composed of viral and cellular proteins in association with the membranous web, a virus-induced organelle composed of remodeled ER membranes. Interestingly, NS5A inhibitors do not inhibit RNA synthesis directly but rather inhibit formation of new RCs [2] in part by blocking biogenesis of the membranous web [4]–a process mediated by the interaction of NS5A with the host lipid kinase phosphatidylinositol-4 kinase IIIα (PI4K-IIIα) [5].

In our previous studies using the genotype 1a H77S.3 virus, we noted a potent but partial inhibition of RNA synthesis by NS5A inhibitors at early time points following addition of antiviral drug to infected cells [2]. In assays that measured viral RNA synthesis, residual viral RNA abundance, and viral polyprotein synthesis, kinetics of antiviral suppression were slower for NS5A inhibitors compared to other classes of DAA such as protease or polymerase inhibitors. Earlier studies using genotype 1b replicon-bearing cells also noted slow kinetics of antiviral activity for NS5A inhibitors compared to other classes of DAA [6]. These data suggest a model where NS5A inhibitors do not block RNA synthesis from pre-existing RCs, but rather prevent formation of new RCs. In contrast to these findings, recent studies using a *Gaussia* luciferase- (GLuc-) expressing virus based on the genotype 2a JFH-1 clone indicated that NS5A inhibitors block GLuc expression faster than protease or polymerase inhibitors [7]. The difference between this and previous studies may be explained by the use of the highly replicative JFH-1.

To better understand the biology underlying the different kinetic responses of JFH-1 and H77S.3 to NS5A inhibitors, we examined a panel of viruses that can replicate in cell culture. Kinetics of reporter virus decline following addition of NS5A inhibitors to infected cell cultures varied according to the sensitivity of the viral RCs to lipid peroxidation (LPO). Lipid peroxidation resistant (LPO^R^) viruses such as JFH-1 [8] and H77D [9] showed a more rapid decline compared to lipid peroxidation sensitive (LPO^S^) viruses such as H77S.3 [10] following treatment with NS5A inhibitors. Further studies capitalized on two isoclonal gt1a cell culture infectious viruses: the LPO^S^ virus H77S.3 and the LPO^R^ virus H77D. To understand differences between LPO^S^ and LPO^R^ viruses, we developed a mathematical model of intracellular RNA dynamics following treatment with DAAs. The different kinetics of decline following NS5A inhibitor treatment can be explained by a model in which the functional RC half-life is shorter for LPO^R^ viruses compared to LPO^S^ viruses. The model was validated using a nascent RNA capture assay to directly monitor the effect of NS5A inhibitors on RNA synthesis. Overall, our results show that the half-life of RCs assembled by different viruses varies widely.

## Results

Our previous studies show that when cells infected with the gt1a HCV clone H77S.3 are cultured with NS5A inhibitors, the decline in intracellular viral RNA abundance and new viral RNA synthesis is relatively slow compared to other classes of DAA such as protease and polymerase inhibitors [2]. These unique kinetics of RNA synthesis inhibition suggested a model where NS5A inhibitors block assembly of new RCs but are unable to inhibit RNA synthesis from pre-existing RCs in infected cells. In contrast to our findings, other studies using reporter viruses based upon the gt2a HCV clone JFH-1 found that NS5A inhibitors resulted in more rapid decline in RNA abundance compared to other classes of DAA [7].

**Fig 1:**
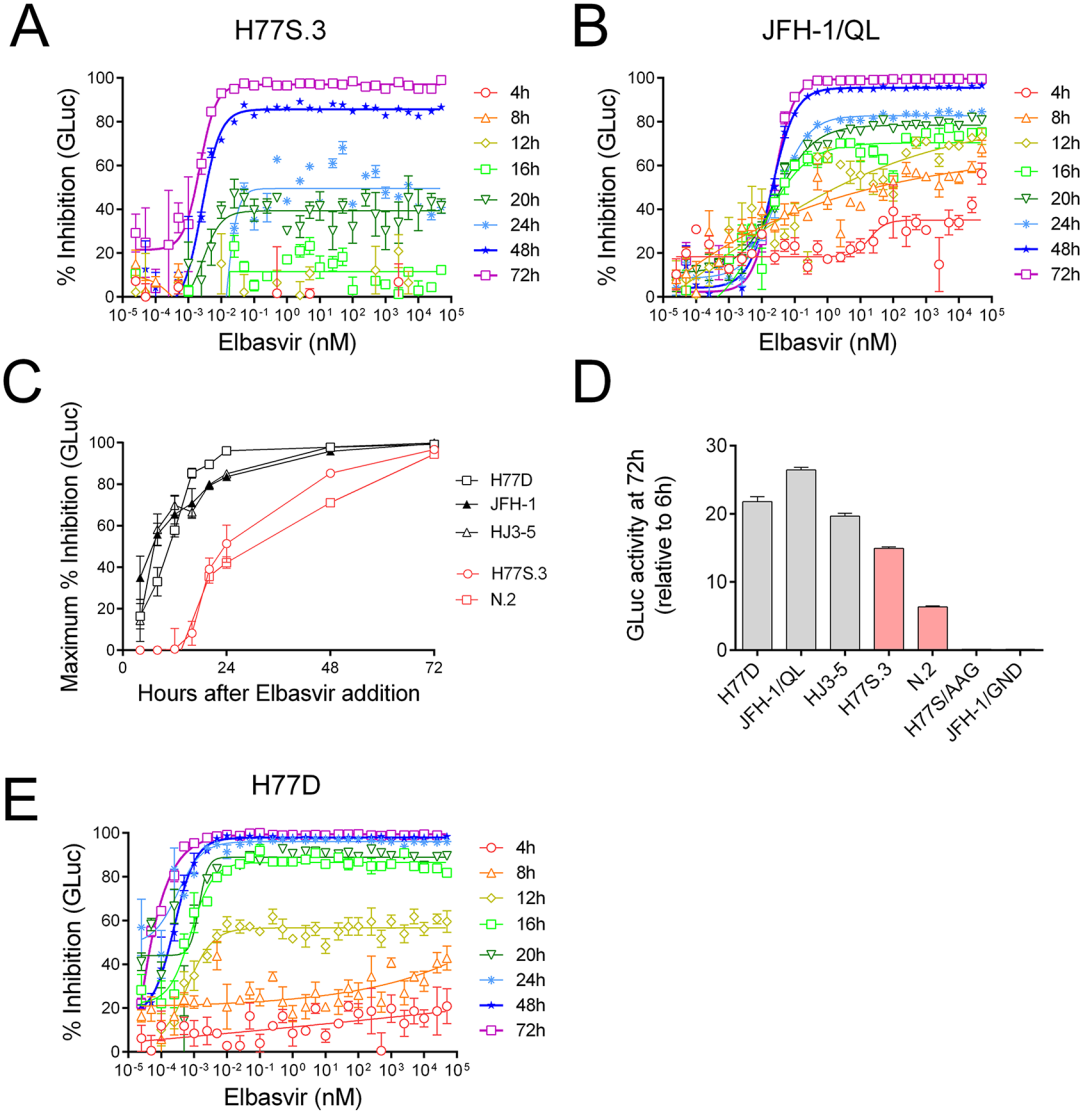
Kinetics of HCV inhibition in infected cell culture following addition of an NS5A inhibitor, monitored using different GLuc reporter viruses. (A) Inhibition of the gt1a LPO^S^ virus H77S.3/GLuc2A by elbasvir. (B) Inhibition of the gt2a LPO^R^ virus JFH-1/QL/GLuc2A by elbasvir. (C) Maximum % inhibition (E_max_) of GLuc activity at different time points after addition of elbasvir to Huh7.5 cells infected with different cell culture infectious HCV genomes. LPO^S^ viruses H77S.3/GLuc2A (gt1a) and N.2/GLuc2A (gt1b) are plotted in red. LPO^R^ viruses H77D/GLuc2A (gt1a), JFH-1/QL/GLuc2A (gt2a) or HJ3-5/GLuc2A (gt1a/2a chimera). (D) Inhibition of the gt1a LPO^R^ virus H77D-GLuc2A by elbasvir. (E) Fitness of different virus genomes used in (C) at 72 h post electroporation. Data shown represents GLuc activity relative to GLuc activity at 6 hours post electroporation to normalize for transfection efficiency. The replication incompetent reporter viruses H77S/AAG/GLuc2A and JFH-1/GND/GLuc2A contain point mutations in the NS5B polymerase and are included as mock-transfection controls.

To resolve this discrepancy, we examined kinetics of antiviral suppression for H77S.3 or JFH-1/QL carrying a GLuc reporter gene. Strikingly different kinetics of antiviral suppression were observed between H77S.3/GLuc2A (Fig 1A) and JFH-1/QL/GLuc2A (Fig 1B) following treatment with the NS5A inhibitor elbasvir. These data suggest that the discrepancies between previous studies [2, 7] represent a genuine difference in the response to NS5A inhibitors between the two viruses and are not simply a result of differences in experimental systems used. The mode of action is likely to be the same for both viruses: NS5A inhibitors block new RC formation but do not block RNA synthesis from pre-existing RCs. The faster kinetics of inhibition for JFH-1/QL/GLuc2A suggest that pre-existing RCs become non-functional faster for JFH-1 compared to H77S.3.

One simple explanation for the difference in kinetics is that JFH-1/QL is genotype 2a whereas H77S.3 is genotype 1a. However, an alternative explanation for the difference in kinetics is differential sensitivity to lipid peroxidation: the JFH-1 replicase is LPO^R^ while the replicase of H77S.3 (and most other HCV clones) is LPO^S^. To distinguish between these possibilities, the kinetics of antiviral suppression by the NS5A inhibitor elbasvir were measured by GLuc assays using a panel of virus genomes including those with LPO^S^ RCs (H77S.3, N.2) and those with LPO^R^ RCs (H77D, HJ3-5, JFH-1/QL) [9]. Differences between the LPO^S^ vs LPO^R^ viruses can be best visualized by plotting the maximum % inhibition (E_max_) against time (Fig 1C). Following addition of elbasvir to infected cell cultures, kinetics of antiviral suppression for LPO^S^ viruses resembled H77S.3 whereas kinetics of antiviral suppression for LPO^R^ viruses resembled JFH-1/QL. Another measure that illustrates the different responses of the two classes of virus to NS5A inhibitor is time until E_max_ = 50% (shown in Table 1).

**Table 1:** Time until E_max_ = 50% for lipid peroxidation sensitive and resistant HCV clones, measured by GLuc assay.

Virus	Lipid peroxidation Sensitive (S) or Resistant (R)	Time (h) until E_max_ = 50% with 95% confidence intervals
H77S.3/GLuc2A	S	22.3 (21.5–23.0)
H77S.3/IS/GLuc2A	S	23.3 (22.2–24.4)
N.2/GLuc2A	S	19.5 (15.0–25.5)
H77D/GLuc2A	R	11.6 (11.2–12.0)
JFH-1/QL/GLuc2A	R	8.9 (7.1–11.1)
HJ3-5/GLuc2A	R	7.5 (7.0–8.0)

Previous studies have suggested that sensitivity to different classes of antivirals and kinetics of antiviral suppression is influenced by replicative fitness [11]. In partial agreement with this study, we find that GLuc expression from highly replicative LPO^R^ virus strains is inhibited faster than for moderately replicative LPO^S^ virus strains (compare kinetics of antiviral suppression in Fig 1C with relative virus fitness measurements in Fig 1D). However, virus fitness is a continuous variable whereas sensitivity or resistance to LPO is a discrete property of the virus-encoded RC. The different virus strains shown in Fig 1C show kinetics of antiviral suppression by elbasvir that fall into two discrete categories: LPO^R^ or LPO^S^.

**Fig 2:**
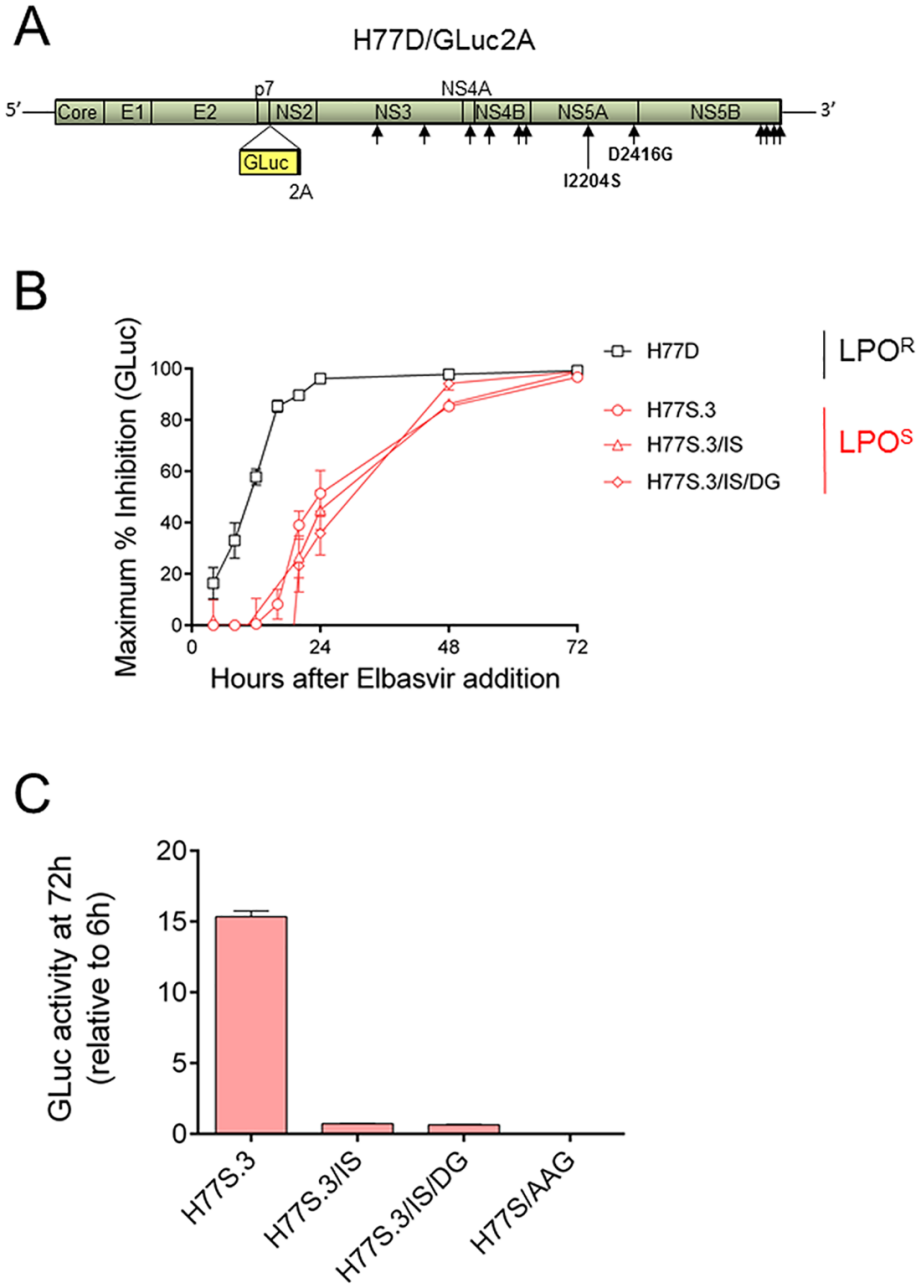
Differences in kinetics of virus inhibition in H77S.3- vs H77D–infected cells following addition of an NS5A inhibitor are not due to differences in NS5A sequence. (A) Diagram of the H77S.3 genome showing positions of the 12 amino acids that differ between H77S.3 and H77D. The two amino acid changes in the NS5A coding region (I2204S and D2416G) are highlighted. (B) Maximum % inhibition (E_max_) at different time points after addition of elbasvir to Huh7.5 cells infected with either H77S.3/GLuc2A, H77D/GLuc2A or H77S.3/GLuc2A carrying either the I2204S mutation alone (H77S.3/IS) or in combination with D2416G (H77S.3/IS/DG). (C) Fitness of different virus genomes used in (B) at 72 h post electroporation. Data shown represents GLuc activity relative to GLuc activity at 6 h post electroporation to normalize for transfection efficiency. The replication incompetent reporter virus H77S/AAG/GLuc2A contains point mutations in the NS5B polymerase and serves as a mock-transfection control.

An intriguing difference in kinetics of inhibition was observed for H77S.3/GLuc2A and H77D/GLuc2A (compare Fig 1A and 1E), two isoclonal genotype 1a viruses. H77D is a LPO^R^ virus derived from H77S.3, a LPO^S^ virus [9]. H77D differs from H77S.3 by only 12 amino acids localized through the non-structural proteins (Fig 2A) but replicates to at least 10-fold higher levels than H77S.3 [9]. Only two of the amino acids that differ between H77S.3 and H77D lie within NS5A: 2204 in low complexity sequence I (LCSI) and 2416 in domain III. Both are distant from the binding site of NS5A inhibitors in domain I. Amino acids 2204 and 2416 (from the N-terminus of the polyprotein) are isoleucine and aspartic acid in H77S.3 and serine and glycine in H77D. Following elbasvir treatment, replication of H77S.3/GLuc2A carrying I2204S and a D2416G mutations in NS5A was suppressed with kinetics similar to H77S.3/GLuc2A, not H77D/GLuc2A (Fig 2B). The kinetics of antiviral suppression were not influenced by differences in fitness resulting from the substitutions (Fig 2C). These data indicate that differences in kinetics of antiviral suppression between H77S.3 and H77D are not caused simply by the differences in NS5A sequence between the two viruses.

The minimum number of adaptive mutations required to convert H77S.3 to a LPO^R^ virus was previously shown to be 8 plus the removal of the key H77S.3 adaptive mutation S2204I in NS5A [9]. The resulting virus is LPO resistant but replicates to levels that are too low to support kinetic analyses of antiviral suppression. An additional compensatory mutation in NS4B (G1909S) confers higher replication levels [9] that permitted kinetic analyses. The LPO^R^ genome H77S.3/IS/8mut/GS declined with kinetics identical to H77D (S1 Fig). Further efforts to dissect the amino acid differences between H77S.3 and H77D showed that changes in NS3, NS4B and NS5B are not responsible for the different kinetics of GLuc decline following NS5A inhibitor treatment (S1 Fig). Previously, detailed analyses showed that an A1672S substitution in NS4A of H77S.3 was a key determinant of resistance to LPO by mutating this position back to alanine from serine in the H77S.3IS/8mut/GS genome shown in Fig 2 [9]. However, introduction of an S1672A substitution into H77S.3IS/8mut/GS results in replication levels that are too low to support detailed kinetic analyses of GLuc. Overall, we conclude that differences in kinetics of GLuc decline following NS5A inhibitor addition required multiple amino acid substitutions in concert (possibly including the A1672S change in NS4A). This is in agreement with previous studies that demonstrated that multiple amino acid substitutions contribute to the LPO sensitivity/resistance phenotype [9]. Also, the difference in kinetics of GLuc decline following NS5A inhibitor addition tracks closely with the LPO sensitivity/resistance phenotype of the replicase. Importantly, the different rates of GLuc activity decline following NS5A inhibitor treatment depend on the combination of amino acid differences across the non-structural proteins that form the replicase.

In cell culture, H77S.3 and other LPO^S^ viruses replicate to lower levels than LPO^R^ viruses such as H77D or JFH-1. Addition of the lipophilic antioxidant vitamin E to cell culture medium can boost replication of H77S.3 by 10-fold but has no effect on H77D [9]. In Fig 1, cells infected with LPO^S^ virus strains were grown in medium containing vitamin E. We hypothesized that the slower rate of decline observed for H77S.3 following NS5A inhibitor treatment was due to a protective effect of vitamin E. However the presence or absence of vitamin E in cell culture medium did not affect the rates of GLuc decline of H77S.3/GLuc2A or H77D/GLuc2A following addition of NS5A inhibitor (S2 Fig). This indicates that RC half-life is not dependent upon the presence or absence of lipid peroxidation itself.

There can be a number of reasons for the different rates of decline in GLuc production by cell cultures infected with the H77S.3/GLuc2A and H77D/GLuc2A viruses following NS5A inhibitor treatment. These include (i) faster packaging and export of H77D genomic RNA from infected cells, (ii) faster turnover of H77D-infected cells, (iii) faster degradation of H77D RNA compared to H77S.3, (iv) faster turnover of functional H77D RCs.

Faster export of H77D is unlikely to be responsible for different rates of GLuc decline following elbasvir treatment since NS5A inhibitors efficiently block virus assembly and release. To formally rule out this possibility, an FFU assay was used to monitor infectious virus production following NS5A inhibitor addition. Rates of inhibition of virus production were not different for H77S.3 and H77D following elbasvir addition (S3 Fig). Thus differences in rate of export of viral RNA from infected cells are not responsible for different rates of GLuc inhibition.

LPO^R^ viruses such as H77D and JFH-1 replicate to higher levels compared to LPO^S^ viruses such as H77S.3 [9]. These higher replication levels result in a greater degree of cell cycle arrest and a higher frequency of apoptosis in infected cells [12]. To explore the possibility that H77D/GLuc2A-infected cells proliferate more slowly or are lost at a faster rate from infected cells cultures, cell viability and proliferation was compared for Huh-7.5 cells following electroporation with H77S.3/GLuc2A, H77D/GLuc2A or the non-replicating H77S/AAG/GLuc2A (S4 Fig). In a WST-1 cell proliferation assay, infected cells grew more slowly compared to mock-infected cells. By 72 h after plating, the lower viability/growth rate of H77D/GLuc2A was apparent by WST-1 assay. Importantly, no difference was observed between the growth rate of cells infected with H77S.3 and H77D at 24 and 48 h after plating. These data suggest that differences in cell viability/proliferation are not responsible for different rates of virus decline following addition of NS5A inhibitors.

**Fig 3:**
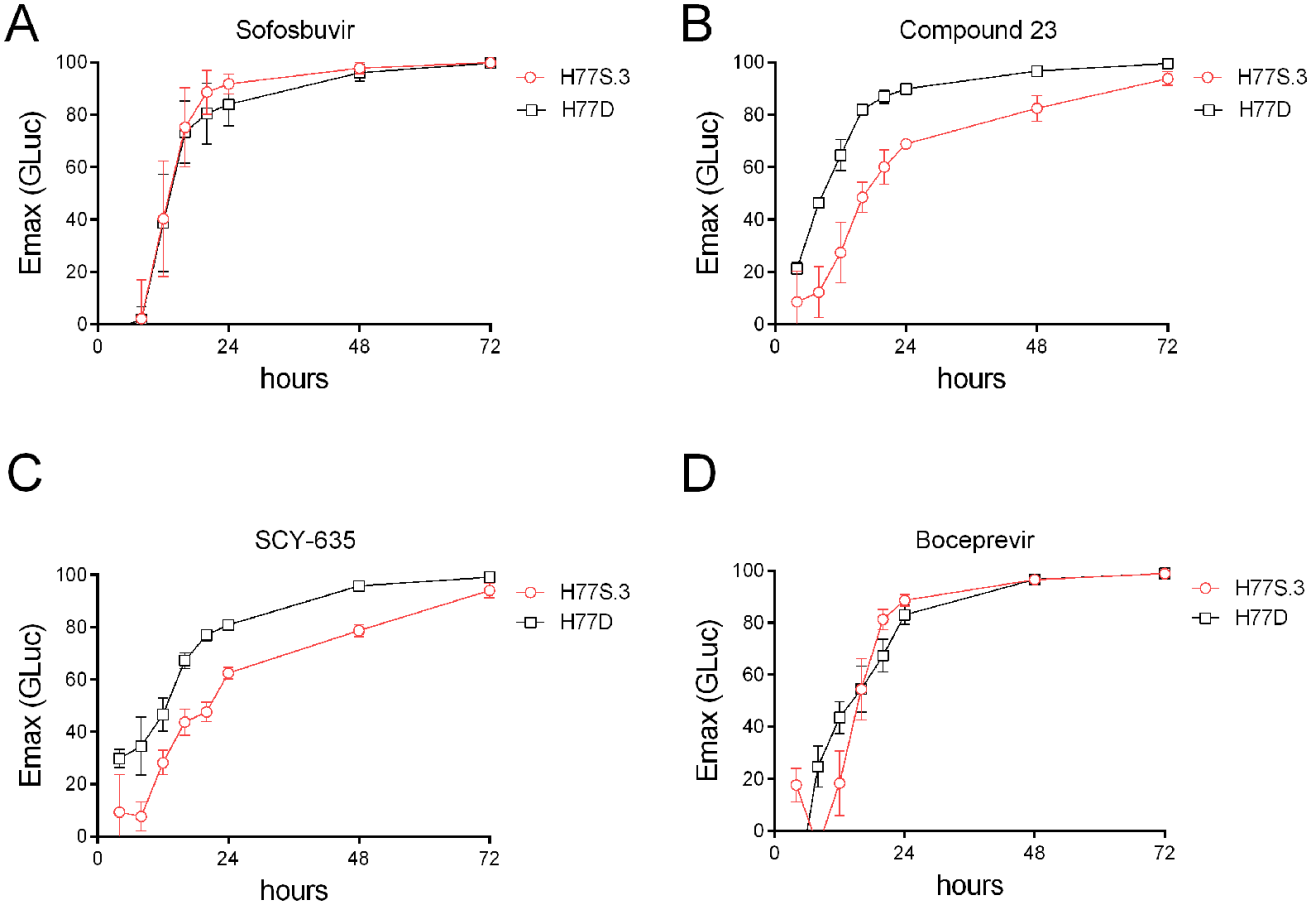
Differences in kinetics of virus inhibition in H77S.3- vs H77D-infected cells are observed with host-targeting antiviral drugs that block membranous web formation but not with the NS5B RdRP inhibitor sofosbuvir or the NS3/4A protease inhibitor boceprevir. Maximum % inhibition (E_max_) at different time points after addition of (A) sofosbuvir, (B) Compound 23: a small molecule inhibitor of PI4KIIIα, (C) SCY-635: a cyclophilin inhibitor, or (D) boceprevir, to Huh7.5 cells infected with H77S.3/GLuc2A or H77D/GLuc2A.

The genomes of H77S.3 and H77D are very similar with only 12 non-synonymous and 1 synonymous nucleotide changes. Nonetheless, it is possible that these changes result in differences in RNA half-life. To examine this possibility, kinetics of GLuc decline following sofosbuvir treatment were measured for both H77D/GLuc2A and H77S.3/GLuc2A. Sofosbuvir is a potent nucleotide inhibitor of the NS5B RdRP and blocks new HCV RNA synthesis so declines in GLuc should be proportional to the rate of (+) strand RNA loss. In the absence of new RNA synthesis, the rate of (+) strand loss from infected cells is determined by a combination of export from the cell and RNA decay, mediated primarily by the cellular 5’ exonuclease Xrn1 [13]. No difference was observed in the kinetics of GLuc decline between the two viruses following addition of sofosbuvir (Fig 3A). In contrast, there was a difference in the rate of GLuc decline between the two viruses following addition of either Compound 23 (Fig 3B), a selective small molecule inhibitor of the host lipid kinase PI4K-IIIα [14], or the cyclophilin inhibitor SCY-635 [15] (Fig 3C). Like NS5A inhibitors, PI4K-IIIα inhibitors and cyclophilin inhibitors both block membranous web formation and as a consequence, inhibit formation of new RCs [11, 16–18]. Although protease inhibitors block formation of new RCs, no difference was observed in the rate of GLuc decline between the H77S.3 and H77D following addition of the PI boceprevir (Fig 3D). This is consistent with previous studies showing that PIs such as boceprevir can block multiple functions of NS3/4A including NS3-dependent functions required for RNA synthesis in preformed replicase complexes [2, 19]. Taken together, these data suggest that differences in kinetics of antiviral suppression by NS5A inhibitors between H77S.3 and H77D reflect differences in half-life of functional RC rather than RNA half-life.

In cell cultures that are infected with GLuc-expressing reporter HCVs, the GLuc activity in the culture medium represents an indirect measure of intracellular RNA abundance. To better understand the biological basis underlying the differences between rates of decline for different viruses following NS5A inhibitor treatment, we developed a mathematical model of RNA dynamics in HCV-infected Huh7.5 cells. To describe the HCV RNA dynamics under different DAAs, we extended previous HCV replication models [3, 20] to consider the dynamics of the replicase complex as well as three key positive strand viral RNA species in the model (see Methods). At the same time, we kept the complexity of the model at a minimum by explicitly considering only the key processes that we study using DAAs (in contrast to previous detailed HCV intracellular models [21]), such that these key processes can be tested and quantified. Analysis of the model showed that the inhibition data of GLuc activity under elbasvir, sofosbuvir and Compound 23 treatment allows a reliable estimate of the half-life of RCs (see S1 Text). We then fitted the model to the inhibition data and found that the model well describes the patterns shown in the data from both H77S.3 and H77D viruses under all three treatments (S5, S6, S7, S8, S9 and S10 Figs). We estimated the rate at which RC becomes nonfunctional and degraded to be 0.20 and 0.07 hour^-1^, corresponding to functional RC half-lives of 3.5 and 9.9 hours, for H77D and H77S.3, respectively (Table 2). For elbasvir treatment, H77S.3 has a higher EC_50_ value than H77D, whereas for sofosbuvir or Compound 23 treatment, the EC_50_ values are estimated to be similar for the two variants (Table 2). Overall, our parameter estimation procedure confirms that the half-lives of functional RCs are drastically different for H77S.3 and H77D variants.

**Table 2:** Descriptions of parameters and their values in the mathematical model.

Fixed parameters	Descriptions	Values	Ref.
*α*	Rate of RNA synthesis	1.7 hour^-1^	[21]
r	Combined rate of replicase complex formation and -RNA synthesis	1 hour^-1^	Fixed*
C_max_	Maximum number of replicase complexes in a cell	40	[27]
W	Rate of GLuc protein translation	1 hour^-1^	[21]
*θ*	Translocation rate of +RNA from the membranous web to the cytosol	0.31 hour^-1^	[21]
*δ*	Degradation rate of +RNA	0.18 hour^-1^	[21]
*K*	Rate of +RNA transport to lipid droplets	1 hour^-1^	Fixed*
*ρ*	Rate of virion assembly and export	0.34 hour^-1^	[3]
d	Degradation rate of GLuc protein	0.35 hour^-1^	[36, 37]
**Fitted parameters**		**Estimated values**	
		**H77D**	**H77S.3**
μ	Rate at which replicase complexes become non-functional and degraded	0.20 hour^-1^	0.07 hour^-1^
σ	Translocation rate of +RNA from the cytosol to the membranous web	0.06 hour^-1^	0.8 hour^-1^
*η*	Translocation rate of +RNA from the site of replication to the ribosome in the cytosol	0.002 hour^-1^	0.09 hour^-1^
*EC*_50,*C*23_	The half maximal response concentration (*EC*_50_) of Compound 23	20.9 nM	18.7 nM
*EC*_50,*ELB*_	The half maximal response concentration (*EC*_50_) of elbasvir	6.0e-05 nM	1.7e-04 nM
*EC*_50,*SOF*_	The half maximal response concentration (*EC*_50_) of sofosbuvir	2447 nM	1812 nM
*τ*_*SOF*_	Pharmacological delay of sofosbuvir	7.6 hour	9.1 hour

* The choice of values of *r* and *k* does not significantly affect the estimation of the rate replicase complexes become non-functional and degraded. They are fixed at rates such that the number of +RNA in each compartment is within biologically plausible ranges.

**Fig 4:**
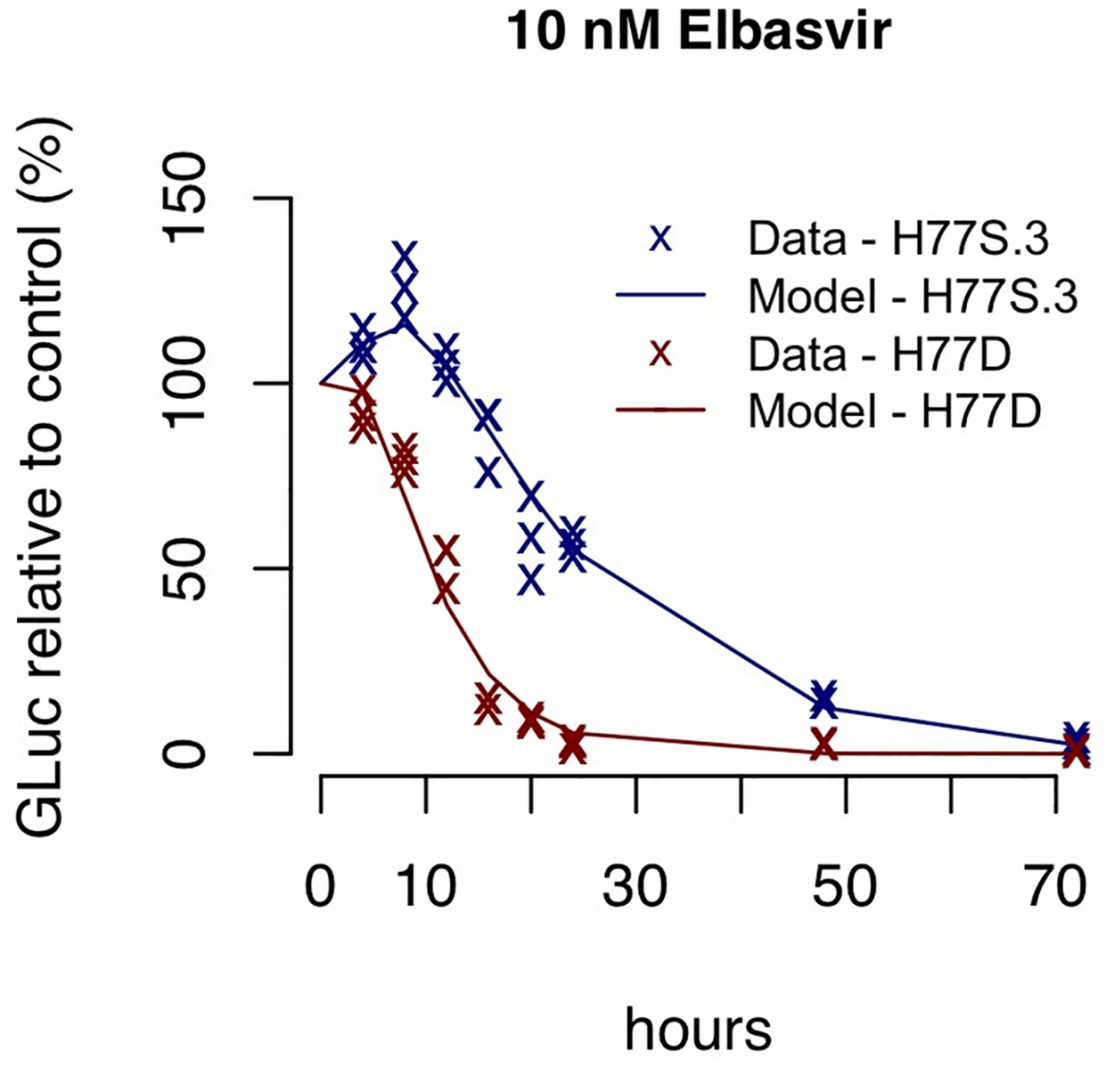
Results of the mathematical model fitted to data measuring the decline of GLuc-reporter activity from the H77S.3 or H77D variants under 10nM elbasvir treatment. Data and simulation using best fit parameter values are shown as ‘x’s and lines, respectively. The data and simulation shown here represent the 10nM elbasvir treatment from S5 and S8 Figs. Here, they are overlaid on the same graph to highlight the transient increase in GLuc activity observed at 4-8h following NS5A inhibitor addition to cell cultures infected with H77S.3/GLuc2A but not H77D/GLuc2A.

There exists a notable delay in the decline in the level of the GLuc-reporter protein upon elbasvir treatment for H77S.3 in contrast to that measured for H77D (Fig 4). Virus particle assembly is rapidly blocked following NS5A inhibitor addition. This results in a transient increase in intracellular RNA levels, since RNA is still being synthesized from preexisting RCs but does not get assembled into virus particles and exported. Modeling results suggest that when H77S.3 assembly is blocked by elbasvir, some of this RNA is redirected from sites of assembly to ribosomes in the cytosol (where they can act as templates for polyprotein synthesis), leading to the transient increase in the H77S.3 GLuc activity immediately upon elbasvir treatment observed in the data (Fig 4 and S5 Fig). In contrast, for H77D, this rate of redirection is estimated to be very low, suggesting H77D +RNAs at blocked sites of assembly may not be redirected to act as templates for polyprotein synthesis. This low rate of redirection combined with a higher rate of RC degradation lead to an immediate decrease in H77D GLuc activity upon elbasvir treatment.

**Fig 5:**
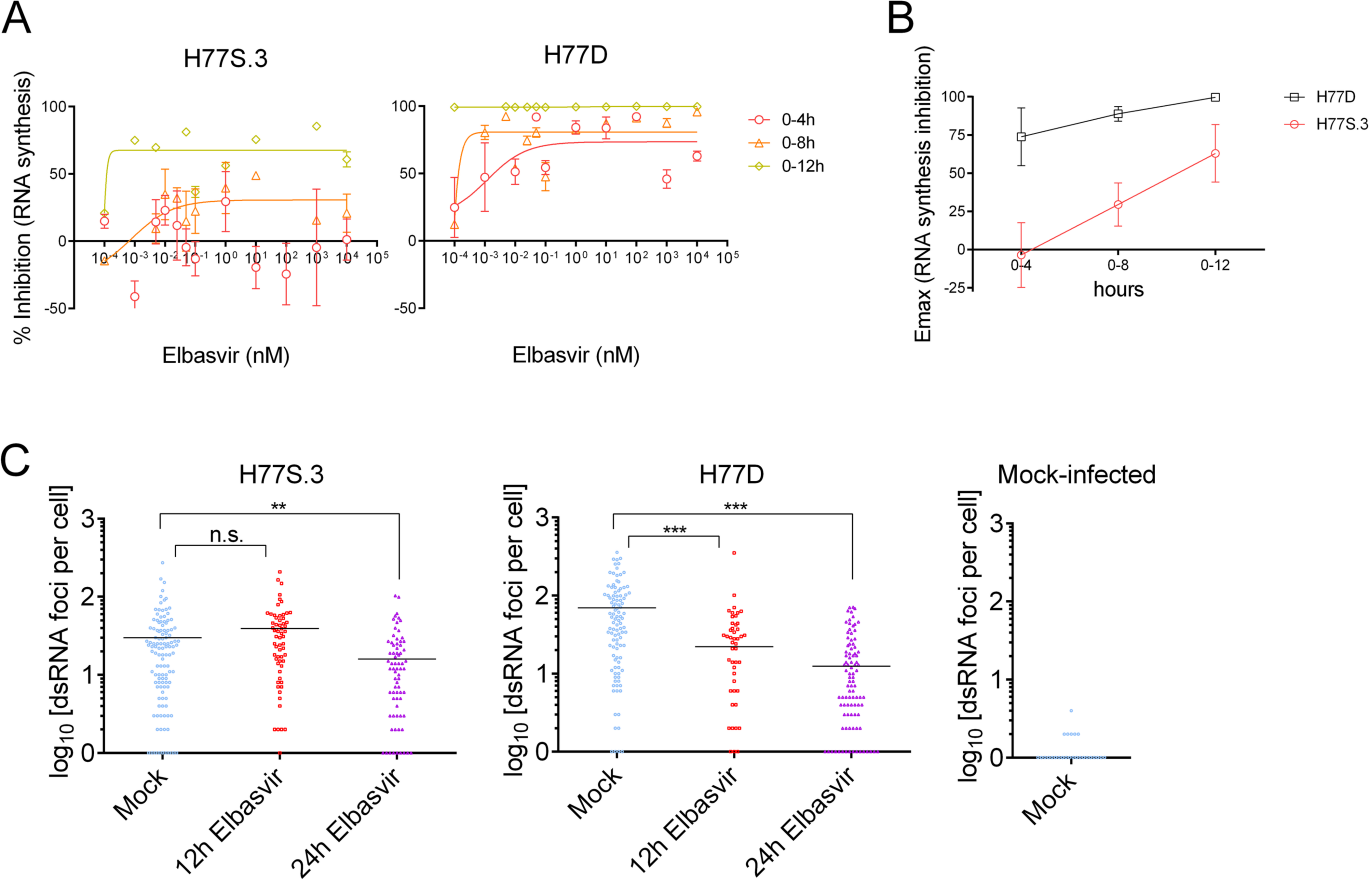
Slower kinetics of RNA synthesis inhibition by an NS5A inhibitor for H77S.3 compared to H77D. (A) Inhibition of RNA synthesis measured in cultures of Huh7.5 cells infected with either H77S.3 (left panel) or H77D (right panel) following treatment with elbasvir. (B) Maximum % inhibition (E_max_) of RNA synthesis at different time points after addition of elbasvir to Huh7.5 cells infected with either H77S.3 or H77D. (C) Measurements of dsRNA foci per cell in Huh7.5 cells infected with either H77S.3 (left panel), H77D (middle panel) or mock-infected cells (right panel). Cells were either mock-treated or treated with 10x EC90 elbasvir for 12 or 24 h before being fixed and stained using a dsRNA specific antibody. Foci of dsRNA per cell were quantified using MetaMorph Image analysis software. Treatment groups were analyzed by Kolmogorov-Smirnoff test (n.s.: not significant, ** p = 0.003, ***p<0.001).

To further test this model and directly monitor decreases in functional RC following NS5A inhibitor treatment, a nascent RNA capture assay combined with qRT-PCR with HCV-specific primers was used. This assay allows measurement of HCV RNA synthesis within different time intervals following addition of DAA. In agreement with our previous studies [2], inhibition of RNA synthesis was potent (i.e., achieved with low drug concentrations) but only partial at early time points following addition of NS5A inhibitor. Importantly, striking differences were observed between H77S.3 and H77D in the rate of decline of RNA synthesis following elbasvir treatment (Fig 5A and 5B). For H77D, elbasvir inhibited new RNA synthesis by greater than 50% within 4 h after addition of drug. In contrast, in H77S.3-infected cells, levels of RNA synthesis inhibition did not exceed 50% until later than 8 h following NS5A inhibitor addition. These measurements strongly support the estimates of RC half-life predicted using the modeling approach.

During HCV RNA synthesis, the positive strand genome acts as a template for a complementary negative strand RNA that can form a double stranded RNA (dsRNA) with the genome. This dsRNA has been proposed to act as a replication intermediate from which new positive strand genomes are synthesized by strand displacement [22]. In HCV-infected cells, dsRNA-containing foci are considered accurate markers for sites of RNA synthesis. Following addition of NS5A inhibitors to HCV-infected cell cultures, the number of dsRNA foci per cell was decreased for both viruses but the number of dsRNA foci per cell declined faster for H77D compared to H77S.3 (Fig 5C). These data further support the conclusion that differences in kinetics of antiviral suppression by NS5A inhibitors between H77S.3 and H77D reflect differences in half-life of functional RC.

## Discussion

Assuming that an NS5A inhibitor binds to NS5A and blocks formation of new membrane-protected RCs soon after its addition to infected cell cultures, the rate of decline of RNA synthesis should reflect the rate of turnover of existing functional RCs. In this study, we have demonstrated that RNA synthesis by LPO^R^ viruses such as H77D show a faster decline in RNA synthesis following treatment with NS5A inhibitors, cyclophilin inhibitors or PI4KIIIα inhibitors compared to LPO^S^ viruses such as H77S.3.

Resistance to LPO is linked to robust viral RNA replication in cell culture. The ability of JFH-1 to grow in cell culture without adaptive mutations is unusual among HCV isolates from patient samples. Only a handful of other HCV isolates have been shown to infect and replicate in cultured cells and have usually required adaptive mutations. For the majority of HCV strains, the RC is highly sensitive to endogenous LPO [9, 23]. Treatment with lipid soluble antioxidants such as vitamin E protects against LPO and boosts RNA replication of most isolates of HCV even those lacking cell culture adaptive mutations. In contrast, the robust replication of JFH-1 is insensitive to LPO and lipid soluble antioxidants. HCV replication can promote LPO through interactions of viral proteins with mitochondria [24]. The sensitivity of the HCV replicase to LPO has been proposed as a mechanism by which the virus can auto-regulate its own replication to limit tissue injury, maintain a reduced immune profile and thus persist in the host [9, 25].

Sensitivity of HCV RNA replication to LPO is complex but maps genetically to several transmembrane or membrane proximal amino acids in NS4A and NS5B. If NS5A inhibitors block formation of new RCs but do not affect preformed RCs, the rate of RNA synthesis decline following addition of inhibitor to infected cells must reflect the decay rate of existing RCs. These data thus suggest that LPO^R^ viruses must have a shorter functional RC half-life compared to LPO^S^ viruses such as H77S.3. It is noteworthy that differences between H77S.3 and H77D in kinetics of RNA synthesis inhibition by NS5A inhibitors are determined by HCV amino acid sequences in transmembrane regions of non-structural proteins. These differences could impact the interaction of non-structural proteins with host membranes to impact the stability of the functional RC. The kinetics of antiviral suppression following addition of elbasvir to cell cultures infected with H77S.3 were not affected by the presence of Vitamin E in the cell culture medium (S2 Fig). This suggests that lipid peroxidation itself does not affect functional RC half-life. Instead the short RC half-life (3.5 h) is an intrinsic feature of the LPO^R^ viruses while the longer RC half-life (9.9 h) is an intrinsic feature of the LPO^S^ viruses. The difference in RC half-life between the LPO^S^ and LPO^R^ viruses is likely determined by amino acid differences in the NS proteins (as shown here for H77S.3 and H77D). It is interesting to speculate that these differences impact the interactions of the NS proteins either with each other, with cellular proteins, or with host membranes in the macromolecular assembly of the RC.

At first glance, it is perhaps surprising that the RCs of robustly replicating viruses (such as H77D or JFH-1 and variants) appear to have a shorter half-life than less replicative viruses (exemplified by H77S.3 but also including most wild type viruses). In infected cells, the HCV RC is localized to the virus-induced organelle known as the membranous web. The membrane structure allows compartmentalization of virus replication and limits recognition of pathogen-associated molecular patterns by host pattern recognition receptors such as RIG-I [26, 27]. There is evidence that the components of the nuclear pore complex are recruited to the membranous web and act to gate access of macromolecules to the component vesicles [26, 28]. Biosynthetic machinery such as ribosomes are excluded from the membranous web [26]. If there is a component of the RC that is limiting and excluded from the lumen of membranous web vesicles after formation, it is possible that each RC can only replicate a fixed number of genomes before that limiting component is exhausted. High replication rates (as observed for H77D and JFH-1) may result in faster depletion of limiting components and a shorter replicase half-life.

If RCs are considered as nano-machines one might expect greater “wear and tear” on highly active machines compared to less active machines. The viral proteins that make up the RC must undergo movements during RNA synthesis (e.g. helicase and polymerase translocating along the RNA template) and such movements are accompanied by dissipative processes or protein friction [29]. This process has been described for protein systems in vitro [30] but the consequences of molecular wear are typically masked by synthesis of new molecules in cellular systems. In HCV infected cells, the membrane-associated RCs are continuously generated at spatially distinct sites [31]. The blockade of membranous web biogenesis by NS5A inhibitors effectively unmasks the decay rate of the membrane-protected HCV RC in infected cells and highlights differences between LPO sensitive and resistant viruses.

## Methods

### Cells, viruses and antiviral drugs

Huh7.5 human hepatoma cells (Apath LLC, Brooklyn, NY) were used for all experiments and maintained in DMEM with 10% FBS, 100U/ml Penicillin/Streptomycin and 1X Glutamax (all Gibco/Life Technologies). Cell culture-infectious viruses used in this study have all been described previously and include: genotype 1a viruses H77S.3 [10] and H77D [9]; the genotype 1b virus N.2 [9]; the genotype 2a virus JFH-1/QL, a derivative of JFH-1 [8, 32] containing a Q221L mutation in the NS3 helicase; the genotype 1a/2a chimera HJ3-5 that encodes the core–NS2 coding sequence of genotype 1a H77S in the background of JFH-1 with compensatory mutations in E1 and NS3 [33]. H77S/AAG is a replication incompetent variant of the cell culture-infectious H77S virus that carries a mutation in the NS5B RNA-dependent RNA polymerase [34]. Viruses carrying a Gaussia luciferase (GLuc) reporter gene have all been described previously and include: H77S.3/GLuc2A [10], H77D/GLuc2A, N.2/GLuc2A, JFH1/QL/GLuc2A and HJ3-5/GLuc2A [9]. Diagrams with the positions of cell culture adaptive mutations in these genomes are shown in S11 Fig. The NS5A inhibitor elbasvir was a gift from Merck Research Laboratories (Kenilworth, NJ). The nucleoside analog NS5B RdRP inhibitor sofosbuvir was purchased from Chemscene (Monmouth Junction, NJ).

### Virus assays

#### Gaussia luciferase assays

Plasmids encoding GLuc reporter virus genomes were linearized by XbaI digestion and transcribed *in vitro* using the T7 Megascript kit (Ambion). RNA products were DNase treated and purified using the RNeasy Mini kit (Qiagen), and electroporated into Huh7.5 cells to initiate virus replication as described previously [10]. At 24 h post electroporation, medium was replaced and cells were cultured for a further 96 h to allow for degradation of input RNA and HCV replication levels to reach a stable maximum. At this time, for both H77S.3 and H77D, >70% of electroporated cells were typically expressing core protein that was detectable by immunofluorescence. These infected Huh7.5 cells were seeded to 96-well plates at 10^4^ cells per well and cultured overnight to allow cells to adhere. Medium was replaced with fresh medium containing inhibitors at a range of concentrations from 100 fM to 50 μM. Medium was harvested for analysis every 4 h and replaced with fresh medium containing DAAs. Cell culture medium was analyzed for GLuc activity using the BioLux Gaussia substrate (New England BioLabs) on a Synergy 2 Multimode Plate Reader (Biotek). Percent inhibition was calculated for each drug concentration and time point. Maximum percent inhibition (Emax) for each DAA was the mean % inhibition for a range of concentrations at the upper asymptotes or plateaux of the dose response curves. The range used depended upon the potency of each DAA. For elbasvir, mean % inhibition for concentrations of 10 nM to 10 μM were used. For Compound 23 and sofosbuvir, mean % inhibition for concentrations ranging from 1–10 μM were used.

#### Cell proliferation assays

For measurement of cell proliferation, WST-1 assay were performed as described previously [12].

#### RNA synthesis assays

To measure kinetics of RNA synthesis suppression by NS5A inhibitors for different viruses (LPO^S^ or LPO^R^), infected cells were incubated with 5-ethynyl uridine (EU) for different intervals (0–4 h, 0–8 h, 0–12 h) after addition of inhibitor. Total RNA was purified from cells using RNeasy minikit (Qiagen) and the newly synthesized EU-labeled RNA was conjugated to biotin and purified from the total RNA using a Click-iT nascent RNA capture kit (Invitrogen). EU-labeled HCV RNA was quantified using a one-step quantitative reverse transcription-PCR assay with primers and probe described previously [10].

#### Immunofluorescence detection of dsRNA foci

HCV-infected Huh7.5 cells, seeded to 4 well chamber slides, were washed twice in 1xPBS, fixed for 30 min in 4% paraformaldehyde and washed twice for 10 min each in 1xPBS. Cells were permeabilized in 0.2% Triton X-100 for 12 minutes, washed twice in PBS-T (1xPBS, 0.1% Tween-20) and blocked for 30 minutes in wash buffer containing 10% goat serum. Cells were incubated with J2 mouse anti-dsRNA monoclonal antibody (Scicons, Budapest, Hungary), diluted 1:200 in PBS-T containing 3% BSA, overnight at 4°C. Cells were washed 3 times for 10 min each in PBS-T. Cells were then incubated in Alexafluor-488-conjugated goat anti-mouse IgG1 (Invitrogen) diluted 1:200 in 3% BSA, PBS-T for 1h at room temperature. Slides were counterstained with DAPI, washed 3 times in PBS-T for 10 minutes each and mounted in Advantage mounting medium (Innovex Biosciences, Richmond, CA).

#### Image analysis

The number of dsRNA foci per cell was quantified using MetaMorph software (Molecular Devices, Sunnyvale, CA). Cell boundaries were estimated by drawing watershed lines between nuclei. Foci of dsRNA were identified based upon size and staining intensity with a minimum threshold of staining intensity that was set based upon images of mock-infected cells (transfected with the replication incompetent RNA genome H77S/AAG, described above).

### Model construction

**Fig 6:**
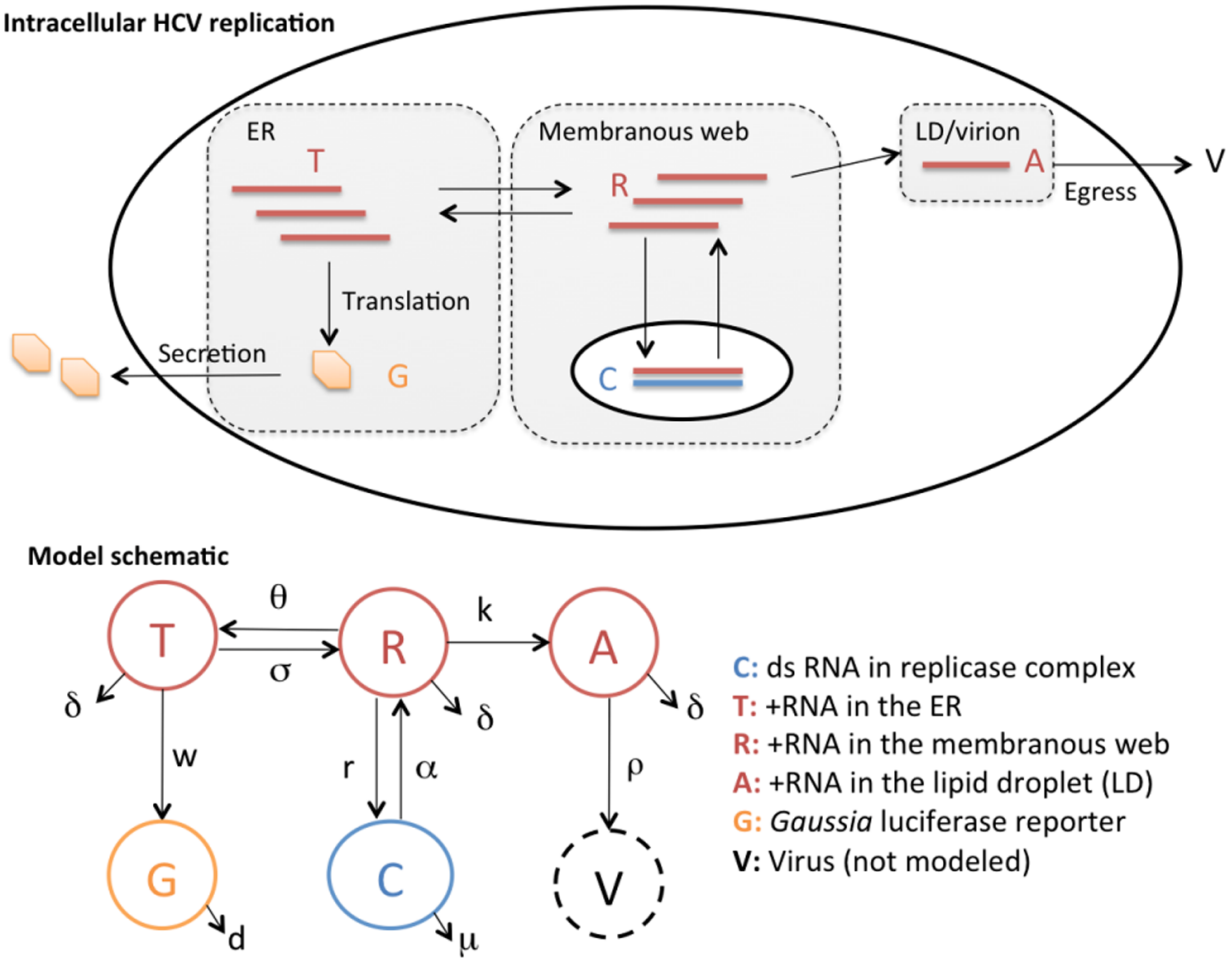
Schematics for intracellular HCV replication dynamics considered in this study and the corresponding mathematical model. We consider the dynamics of positive strand HCV RNAs (+RNAs) in the ER (T), in the membranous web (R) and in the lipid droplets (A), the replicase complex (C) and the extracellular *Gaussia* luciferase (GLuc) proteins (G). See text for detailed explanations.

We developed an ordinary differential equation (ODE) model to keep track of the dynamics of positive strand HCV RNAs (+RNAs) that are available for translation in the cytosol/ER (*T*), +RNAs available for replication in the membranous web (*R*), or +RNAs associated with the lipid droplets for assembly and export (*A*). We also keep track of the dynamics of the replicase complex (*C*). The amount of extracellular *Gaussia* luciferase (GLuc) protein is represented by *G*. See Fig 6 for a schematic. The following equations describe the model:

$$\frac{dT}{dt}=\theta R-\sigma T+\eta A-\delta T$$

$$\frac{dR}{dt}=\sigma T-\theta R-\left(1-\varepsilon_{SOF}\right)\left(1-\varepsilon_{ELB}\right)\left(1-\varepsilon_{C23}\right)r\left(1-\frac{C}{C_{max}}\right)R+\left(1-\varepsilon_{SOF}\right)\alpha C-kR-\delta R$$

$$\frac{dC}{dt}=\left(1-\varepsilon_{SOF}\right)\left(1-\varepsilon_{ELB}\right)\left(1-\varepsilon_{C23}\right)r\left(1-\frac{C}{C_{max}}\right)R-\mu C$$

$$\frac{dA}{dt}=kR-\eta A-\delta A-\left(1-\varepsilon_{ELB}\right)\rho A$$

$$\frac{dG}{dt}=wT-dG$$

$$\varepsilon_{D}=\frac{\left[D\right]}{EC_{50,D}+\left[D\right]}$$

where *D* ∈ {*ELB*, *SOF*, *C*23}

In this model, +RNAs are transported from the cytosol/ER to the membranous web at rate *σ* and from the membranous web (*R*) to the cytosol/ER (*T*) at rate *θ*. +RNAs associated with the lipid droplets (*A*) can dissociate from the lipid droplets and be transported to the cytosol/ER to serve as templates for translation at rate *η*. The +RNAs in the membranous web (*R*) associate with NS5A and other viral and host proteins to form the replicase complexes (*C*). Within the replicase complex, negative strand RNAs (-RNAs) are synthesized to form double-strand HCV RNAs and then serves as templates for +RNA synthesis. The formation of replicase complex and RNA synthesis are assumed to occur at rate *r*. Since there is a maximum number of replicase complexes that can be formed in a cell [27], we use the term $r\left(1-\frac{C}{C_{max}}\right)$ to describe the rate of replicase complex formation, where *C*_*max*_ is the carrying capacity of replicase complexes. +RNAs (*R*) are produced from the replicase complex at rate *α*, and then they can be shuttled to lipid droplets (to become *A*) at rate *k*. The +RNAs associated with the lipid droplets (*A*) are assembled and packaged into virions and exported extracellularly at rate *ρ*. For simplicity, we assume that all +RNAs degrade at rate *δ* and the replicase complex becomes non-functional and degraded at rate *μ*. GLuc proteins are translated from +RNAs and secreted into the extracellular medium at rate *w*, and are degraded at rate *d*.

The impact of DAAs are modeled using the term (1 − *ε*_*D*_), where *D* represents the NS5A inhibitor elbasvir (ELB), the nucleotide analog sofosbuvir (SOF) or Compound 23 (C23), and *ε*_*D*_ is the efficacy of the drug treatment at blocking its targeted processes. Elbasvir blocks the formation of new replicase complex (the process modeled by the term $r\left(1-\frac{C}{C_{max}}\right)R$) and viral assembly (the process modeled by the term *ρA*), whereas Compound 23 only blocks the formation of new replicase complexes. Sofosbuvir blocks the synthesis of both + and—strand HCV RNAs, which are described by the terms *αC* and $r\left(1-\frac{C}{C_{max}}\right)R$, respectively.

We use an Emax model to describe the relationship between the drug concentration, [*D*], and its efficacy: $\varepsilon_{D}=\frac{\left[D\right]}{EC_{50,D}+\left[D\right]}$, where *EC*_50,*D*_ is the drug concentration needed for a half maximal response of an HCV strain. In addition, we assume that there is a pharmacological delay in the action of sofosbuvir [35], *τ*_*SOF*_: the effective drug concentration is 0 when *t* < *τ*_*SOF*_, and it becomes the concentration used in the experiment when *t* > *τ*_*SOF*_.

#### Simulation and data fitting

To simulate the model, we first run the model without drug treatment so that the model reaches its non-zero steady-state. We then run four sets of simulations: one without treatment and then ones with elbasvir, sofosbuvir and Compound 23 treatment. At each time point of data collection under a drug treatment, the percentage of inhibition is calculated as the percentage ratio of the level of the GLuc protein in the treated simulation over the level in the untreated simulation. The level of GLuc protein is then set to 0 at each time point of data collection in the model to reflect the harvesting of medium in the experimental procedure.

To estimate key parameters in the model, we fixed the values of many parameters according to estimates reported previously and estimated the values of three rate constants, *μ*, *σ* and *η*, as well as three pharmacokinetics parameters, EC_50,ELB_, EC_50,SOF_ and *τ*_*SOF*_(Table 2). We derived analytical results (Supporting Information) to confirm that, under high concentrations of drug treatment, the long-term decline of GLuc protein levels under elbasvir and Compound 23 treatments are both driven by the rate of the replicase complex become nonfunctional and degraded, *μ*, whereas the long-term decline under sofosbuvir treatment is determined by a combination of those transport rate constants (*σ*, *θ*, *η*, *k* and *ρ*) and degradation rate constants (*δ* and *μ*).

Parameter estimation is performed by minimizing the residual sum of squares (RSS) between model prediction and data. RSS for a set of parameter values is calculated by summing the squared differences between model predicted percentages of inhibition as described above for all three drug treatments and their corresponding experimental measurements. In this way, each set of estimated parameter values simultaneously describes the GLuc inhibition data under elbasvir, sofosbuvir and Compound 23 treatments.

## Supporting information

S1 Fig Differences in kinetics of viral decline in H77S.3- vs H77D–infected cells following addition of an NS5A inhibitor are determined by a combination of amino acid differences across the entire NS proteins rather than differences in specific NS proteins. (A) Diagram of the H77S.3 mutant genomes showing positions of the amino acids that were changed to H77D: (i) H77S.3/IS/8mut/GS contains 8 mutations previously shown to confer resistance to LPO together with the I2204S mutation in NS5A, and the NS4B compensatory mutation G1909S (ii) H77S.3/IS/AG/FL contains two mutations in NS3 (A1226G and F1464L); (iii) H77S.3/IS/QH/NT contains two mutations in NS4B (Q1773H and N1927T); (iv) H77S.3/IS/DG/YF/NS contains three mutations in NS5B (D2979G, Y2981F and F2994S). Additionally, all of these genomes contain the I2204S mutation in NS5A, which is required for compatibility of the other mutations with the H77S.3 background. (B) Maximum % inhibition (E_max_) at different time points after addition of elbasvir to Huh7.5 cells infected with either H77S.3, H77D or H77S.3 mutants depicted in panel (A).
(TIF)

S2 Fig Addition of the lipophilic antioxidant vitamin E to cell culture medium does not change rate of decline of intracellular RNA abundance for H77S.3 or H77D following addition of elbasvir to infected cell cultures. Maximum % inhibition (E_max_) at different time points after addition of elbasvir to Huh7.5 cells infected with H77S.3/GLuc2A or H77D/GLuc2A that were maintained in the presence or absence of 1μM vitamin E.
(TIF)

S3 Fig No difference in inhibition of virus production (assembly and release) by elbasvir (10X EC90 concentration) in cells infected with H77S.3 or H77D. (TIF)

S4 Fig Cell proliferation measured by WST-1 assay. Huh7.5 cells were electroporated with either H77S.3/GLuc or H77D/GLuc to initiate virus replication or replication incompetent H77S/AAG/GLuc as an uninfected control. Cells were cultured for 3 days prior to seeding to 96 well plates at 1x10^5^ cells per well as for a GLuc assay. Cell proliferation was measured by WST-1 assay at 24, 48 and 72 hours after plating. Viability and proliferation of cells infected with H77S.3 and H77D were compared by unpaired, two-tailed t-test (n.s.: not significant; * P = 0.0042; ***P<0.0001).
(TIF)

S5 Fig Fitting results of the mathematical model to data measuring the percentage reduction of GLuc-reporter activity from the H77S.3 strain under elbasvir treatment. Data and simulation using best fit parameter values are shown as ‘x’s and lines, respectively. The drug concentration is shown as the title of each subplot. The data presented in this figure is the same set of data shown in Fig 1A.
(TIF)

S6 Fig Fitting results of the mathematical model to data measuring the percentage reduction of GLuc-reporter activity from the H77S.3 strain under sofosbuvir treatment. Data and simulation using best fit parameter values are shown as ‘x’s and lines, respectively. The drug concentration is shown as the title of each subplot.
(TIF)

S7 Fig Fitting results of the mathematical model to data measuring the percentage reduction of GLuc-reporter activity from the H77S.3 strain under Compound 23 treatment. Data and simulation using best fit parameter values are shown as ‘x’s and lines, respectively. The drug concentration is shown as the title of each subplot.
(TIF)

S8 Fig Fitting results of the mathematical model to data measuring the percentage reduction of GLuc-reporter activity from the H77D strain under elbasvir treatment. Data and simulation using best fit parameter values are shown as ‘x’s and lines, respectively. The drug concentration is shown as the title of each subplot. The data presented in this figure is the same set of data shown in Fig 1D.
(TIF)

S9 Fig Fitting results of the mathematical model to data measuring the percentage reduction of GLuc-reporter activity from the H77D strain under sofosbuvir treatment. Data and simulation using best fit parameter values are shown as ‘x’s and lines, respectively. The drug concentration is shown as the title of each subplot.
(TIF)

S10 Fig Fitting results of the mathematical model to data measuring the percentage reduction of GLuc-reporter activity from the H77D strain under Compound 23 treatment. Data and simulation using best fit parameter values are shown as ‘x’s and lines, respectively. The drug concentration is shown as the title of each subplot.
(TIF)

S11 Fig Diagrams of HCV genomes carrying GLuc reporter genes used in experiments shown in Fig 1. (A) H77S.3/GLuc2A, derived from gt 1a strain H77, Genbank Accession AF011751, and containing the indicated cell culture adaptive mutations. (B) H77D/GLuc2A, derived from H77S.3/GLuc2A. Amino acid positions that differ from H77S.3/GLuc2A are indicated. All are cell culture adaptive mutations except I2204S, shown red, which is mutated back to the original H77 sequence. (C) N.2/GLuc2A, derived from gt1b HCV-N strain, Genbank Accession AF139594, and containing the indicated cell culture adaptive mutations. (D) HJ3-5/GLuc2A, a chimeric gt1a/2a virus containing core to NS2 sequence from H77 and NS3 to NS5B sequence from JFH-1 with compensatory mutations in E1 and NS3. (E) JFH-1/QL/GLuc2A, derived from gt2a strain JFH-1, Genbank Accession AB047639, and containing the indicated cell culture adaptive mutation.
(TIF)

S1 Text Analytical approximations to the mathematical model of intracellular HCV RNA dynamics. (DOCX)
