# Supplementary figures and images for: NS5A inhibitors unmask differences in functional replicase complex half-life between different hepatitis C virus strains
Source: PLoS Pathog. 2017 Jun 8;13(6):e1006343. doi: 10.1371/journal.ppat.1006343 (PMC5464671; doi:10.1371/journal.ppat.1006343)

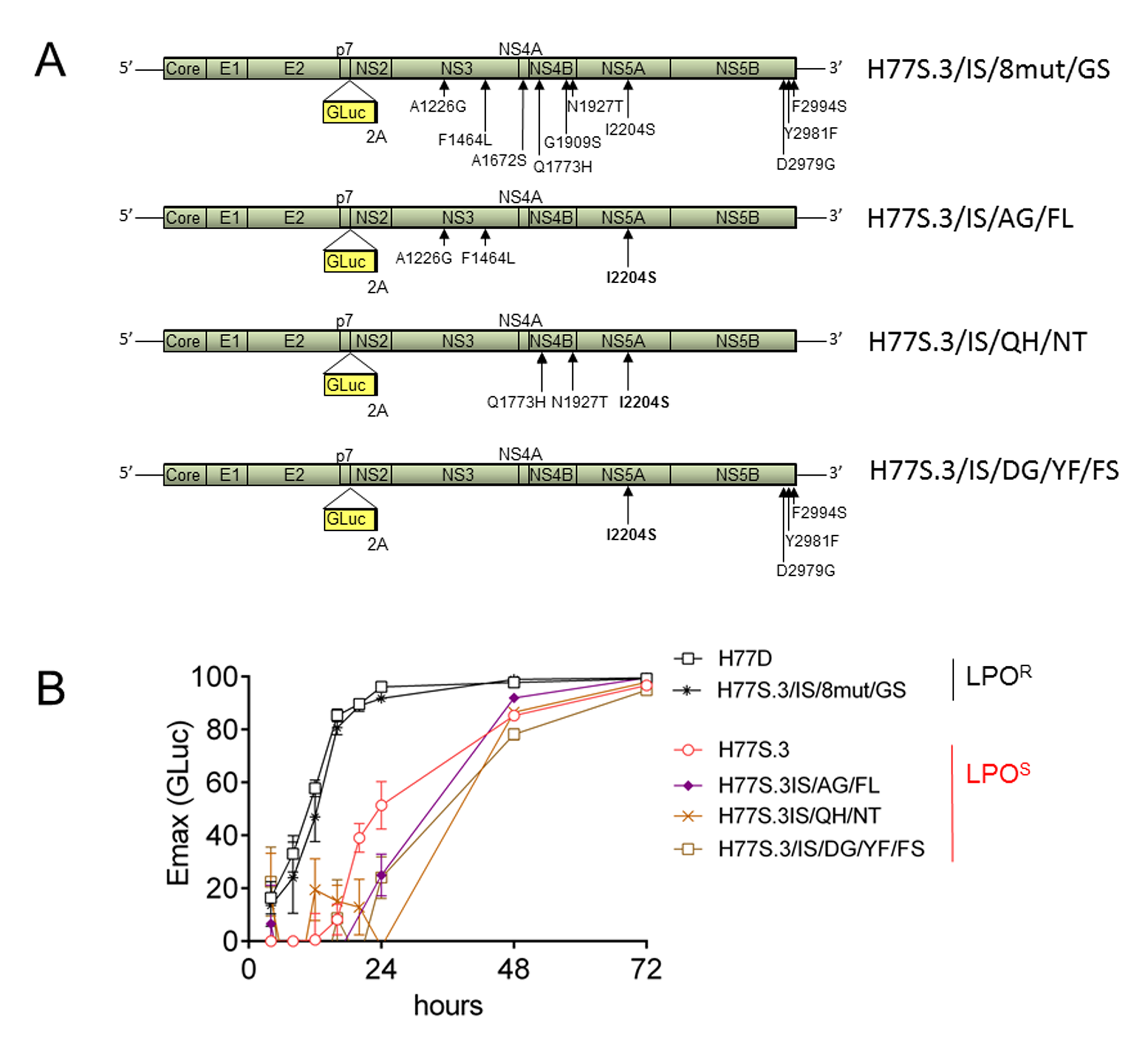

Supplement: S1 Fig — (A) Diagram of the H77S.3 mutant genomes showing positions of the amino acids that were changed to H77D: (i) H77S.3/IS/8mut/GS contains 8 mutations previously shown to confer resistance to LPO together with the I2204S mutation in NS5A, and the NS4B compensatory mutation G1909S (ii) H77S.3/IS/AG/FL contains two mutations in NS3 (A1226G and F1464L); (iii) H77S.3/IS/QH/NT contains two mutations in NS4B (Q1773H and N1927T); (iv) H77S.3/IS/DG/YF/NS contains three mutations in NS5B (D2979G, Y2981F and F2994S). Additionally, all of these genomes contain the I2204S mutation in NS5A, which is required for compatibility of the other mutations with the H77S.3 background. (B) Maximum % inhibition (Emax) at different time points after addition of elbasvir to Huh7.5 cells infected with either H77S.3, H77D or H77S.3 mutants depicted in panel (A). (TIF) [file ppat.1006343.s001.tif]

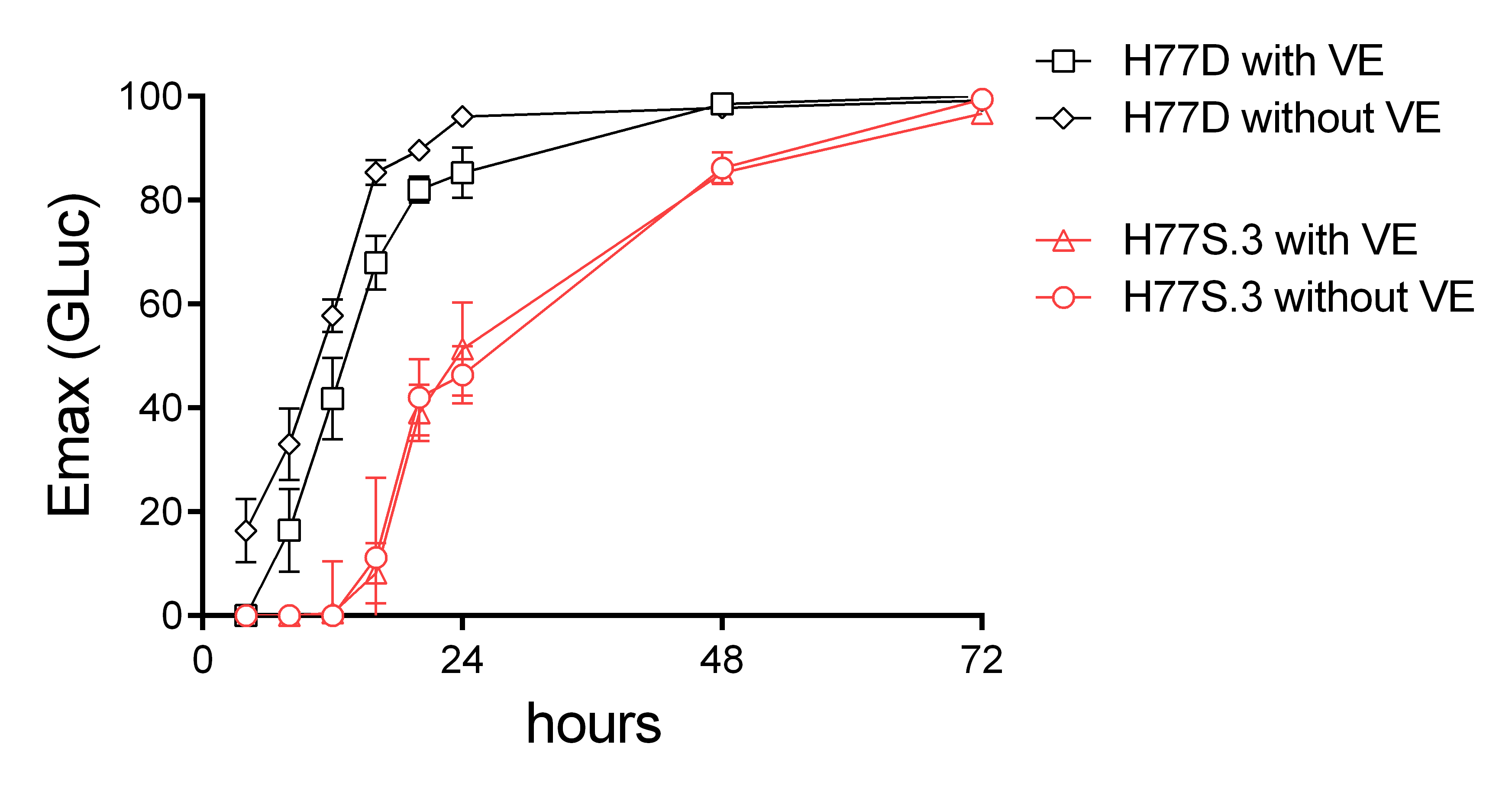

Supplement: S2 Fig — Maximum % inhibition (Emax) at different time points after addition of elbasvir to Huh7.5 cells infected with H77S.3/GLuc2A or H77D/GLuc2A that were maintained in the presence or absence of 1μM vitamin E. (TIF) [file ppat.1006343.s002.tif]

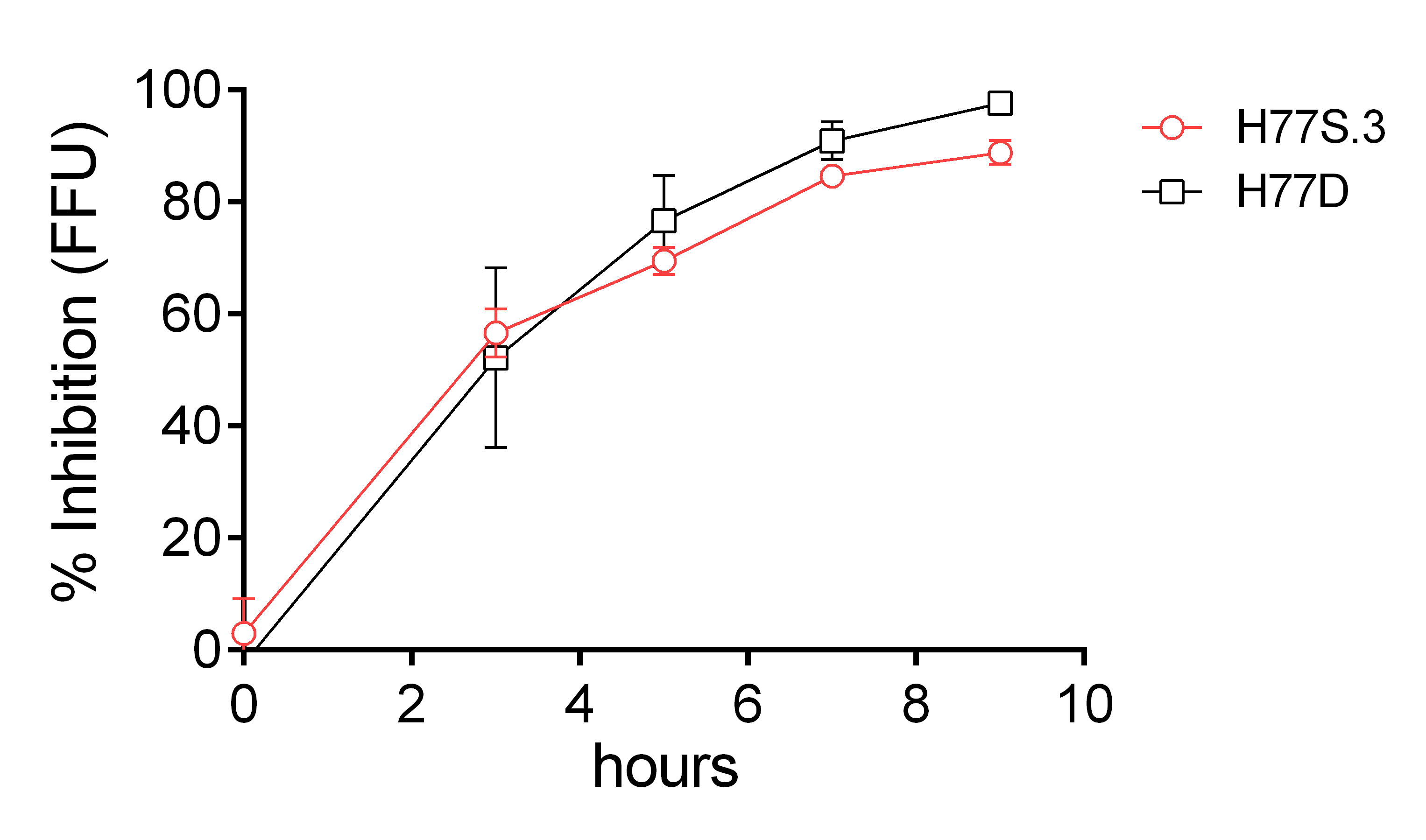

Supplement: S3 Fig — (TIF) [file ppat.1006343.s003.tif]

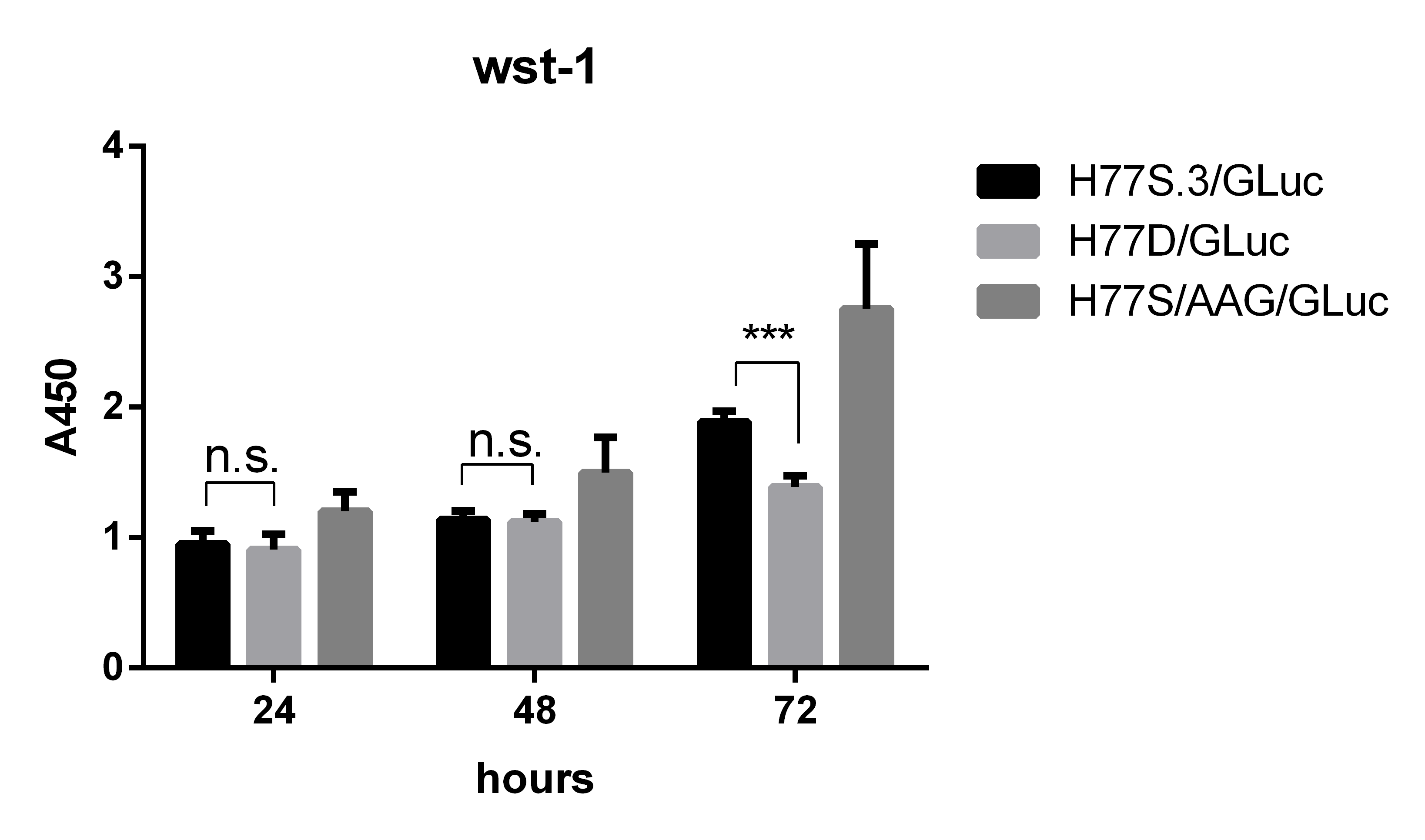

Supplement: S4 Fig — Huh7.5 cells were electroporated with either H77S.3/GLuc or H77D/GLuc to initiate virus replication or replication incompetent H77S/AAG/GLuc as an uninfected control. Cells were cultured for 3 days prior to seeding to 96 well plates at 1x10^5^ cells per well as for a GLuc assay. Cell proliferation was measured by WST-1 assay at 24, 48 and 72 hours after plating. Viability and proliferation of cells infected with H77S.3 and H77D were compared by unpaired, two-tailed t-test (n.s.: not significant; * P = 0.0042; ***P<0.0001). (TIF) [file ppat.1006343.s004.tif]

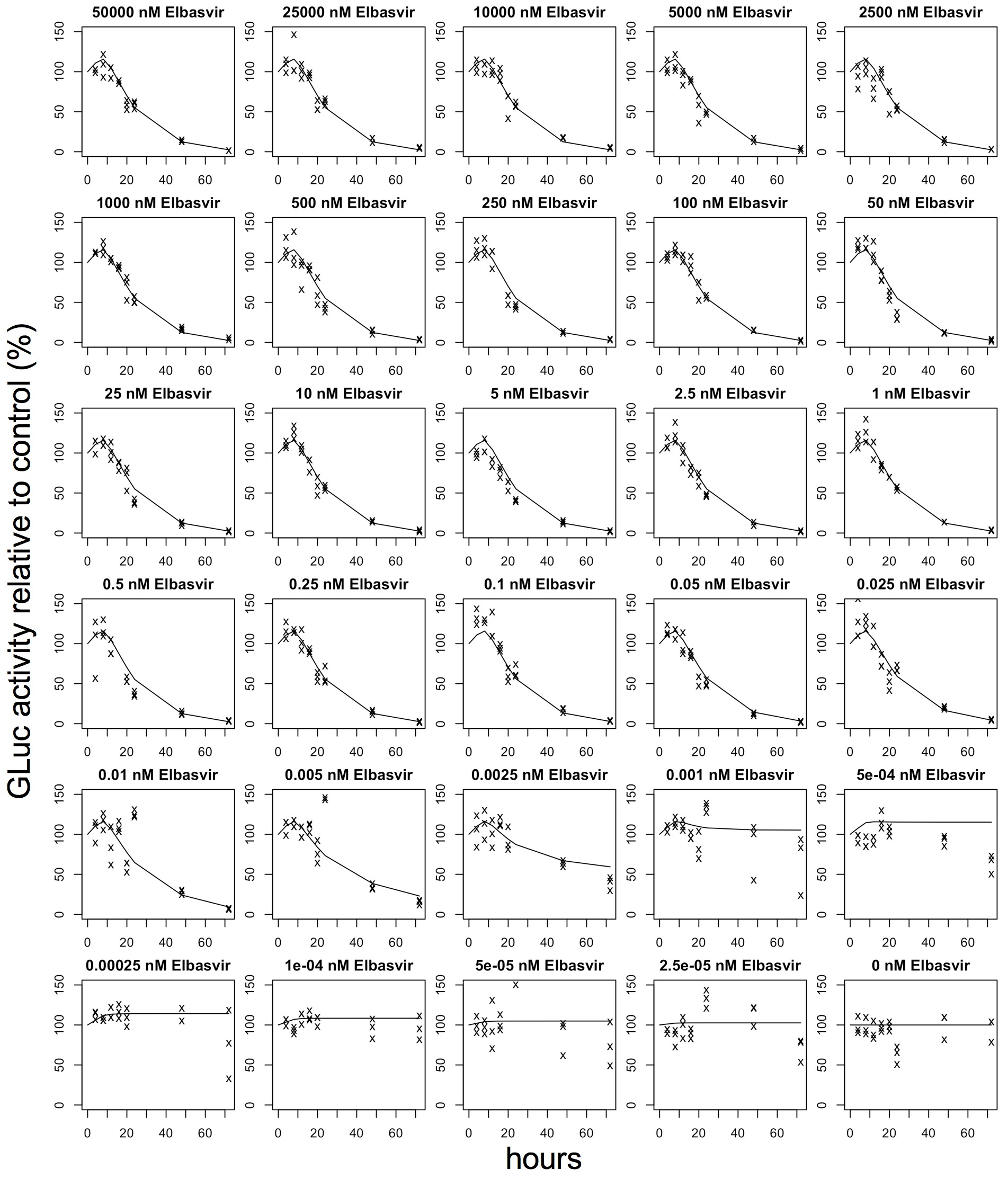

Supplement: S5 Fig — Data and simulation using best fit parameter values are shown as ‘x’s and lines, respectively. The drug concentration is shown as the title of each subplot. The data presented in this figure is the same set of data shown in Fig 1A. (TIF) [file ppat.1006343.s005.tif]

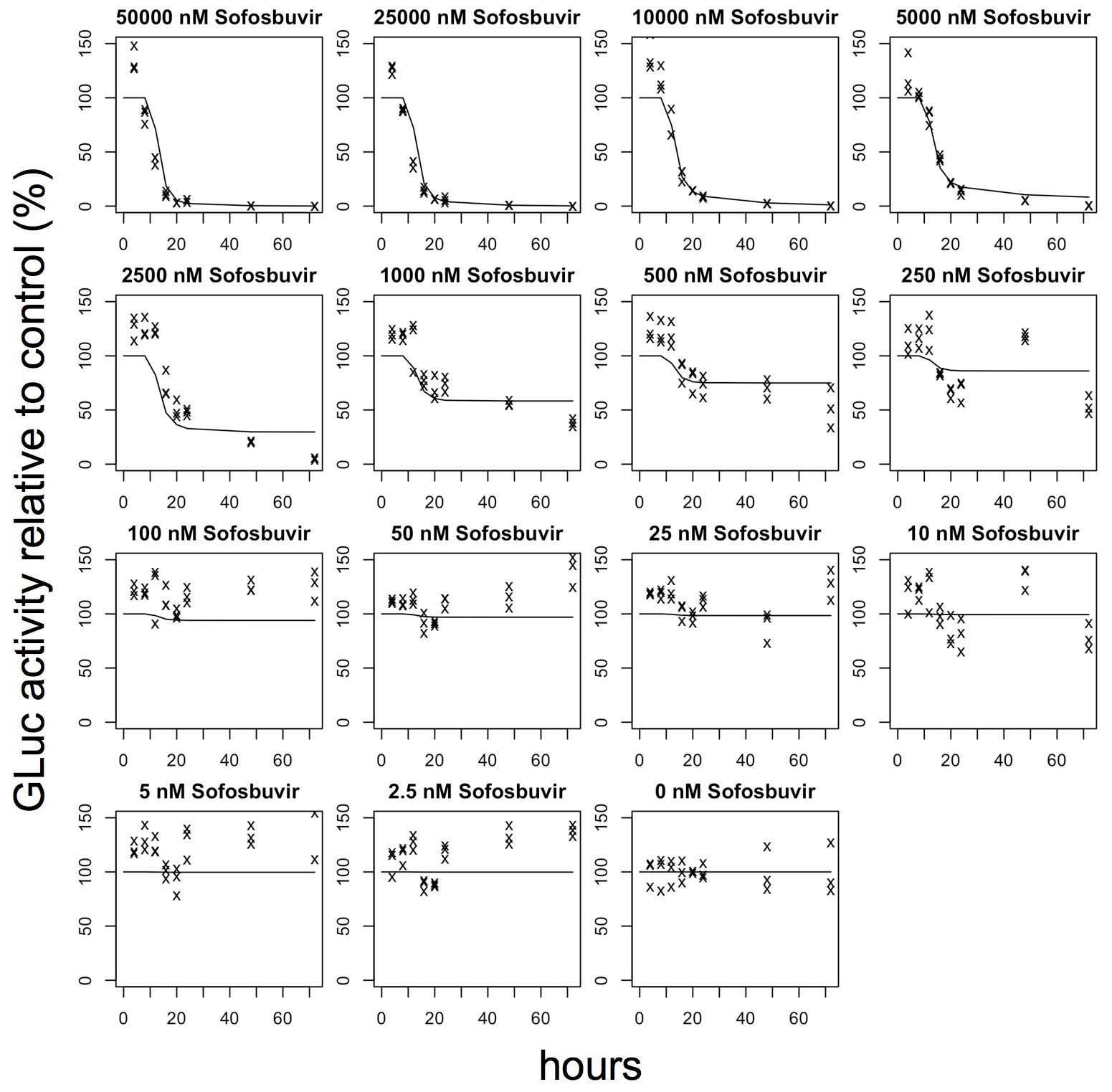

Supplement: S6 Fig — Data and simulation using best fit parameter values are shown as ‘x’s and lines, respectively. The drug concentration is shown as the title of each subplot. (TIF) [file ppat.1006343.s006.tif]

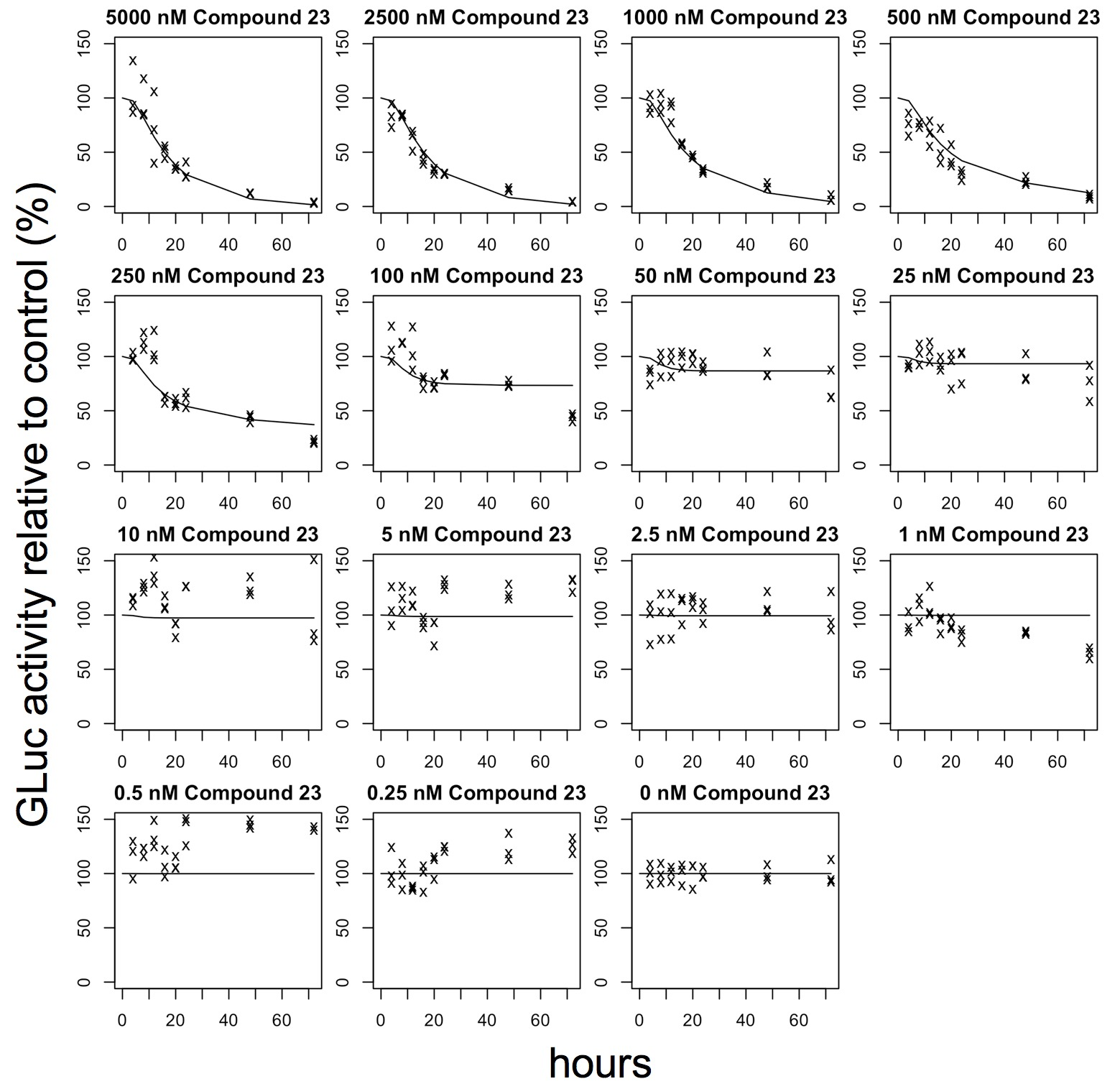

Supplement: S7 Fig — Data and simulation using best fit parameter values are shown as ‘x’s and lines, respectively. The drug concentration is shown as the title of each subplot. (TIF) [file ppat.1006343.s007.tif]

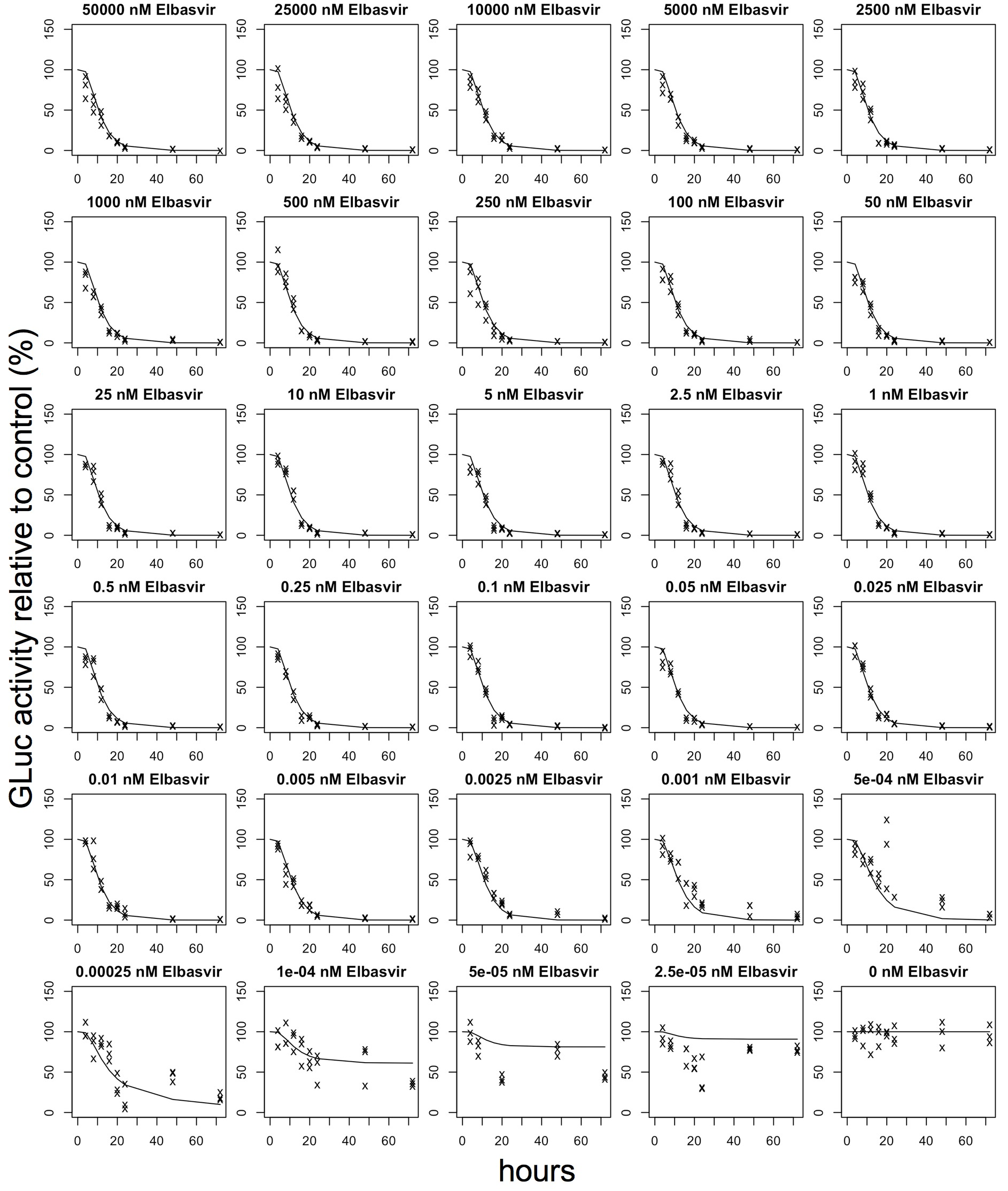

Supplement: S8 Fig — Data and simulation using best fit parameter values are shown as ‘x’s and lines, respectively. The drug concentration is shown as the title of each subplot. The data presented in this figure is the same set of data shown in Fig 1D. (TIF) [file ppat.1006343.s008.tif]

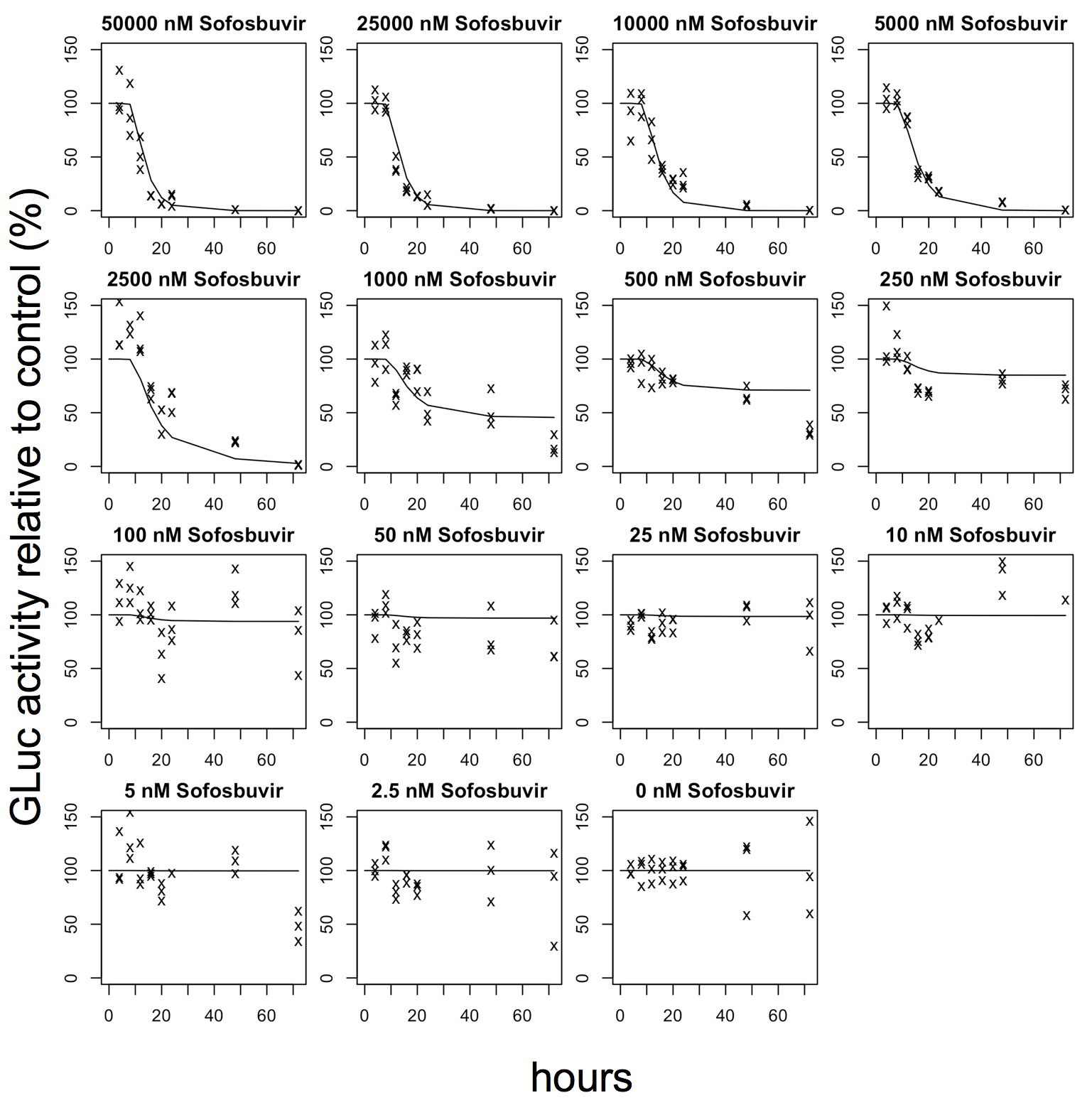

Supplement: S9 Fig — Data and simulation using best fit parameter values are shown as ‘x’s and lines, respectively. The drug concentration is shown as the title of each subplot. (TIF) [file ppat.1006343.s009.tif]

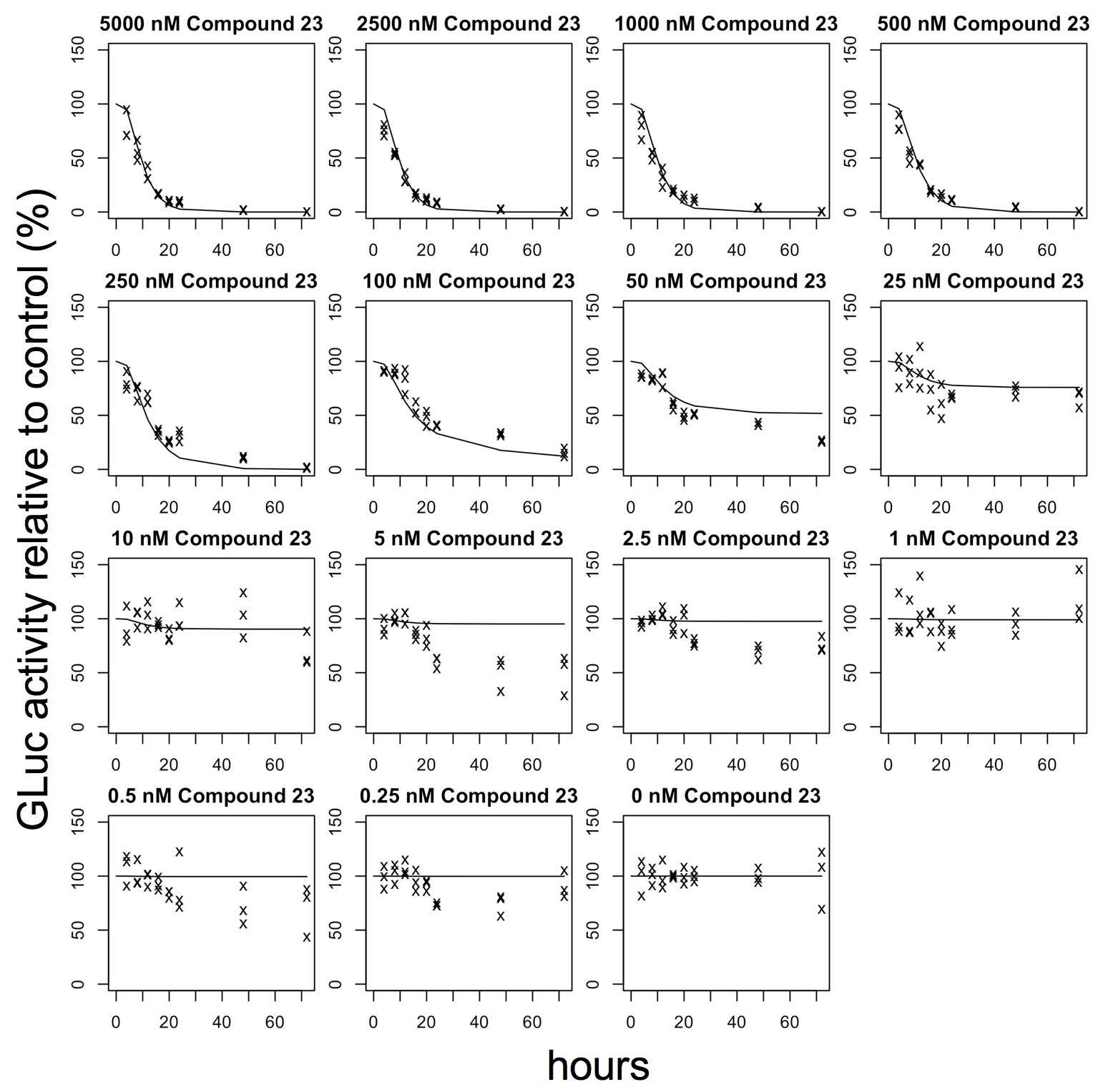

Supplement: S10 Fig — Data and simulation using best fit parameter values are shown as ‘x’s and lines, respectively. The drug concentration is shown as the title of each subplot. (TIF) [file ppat.1006343.s010.tif]

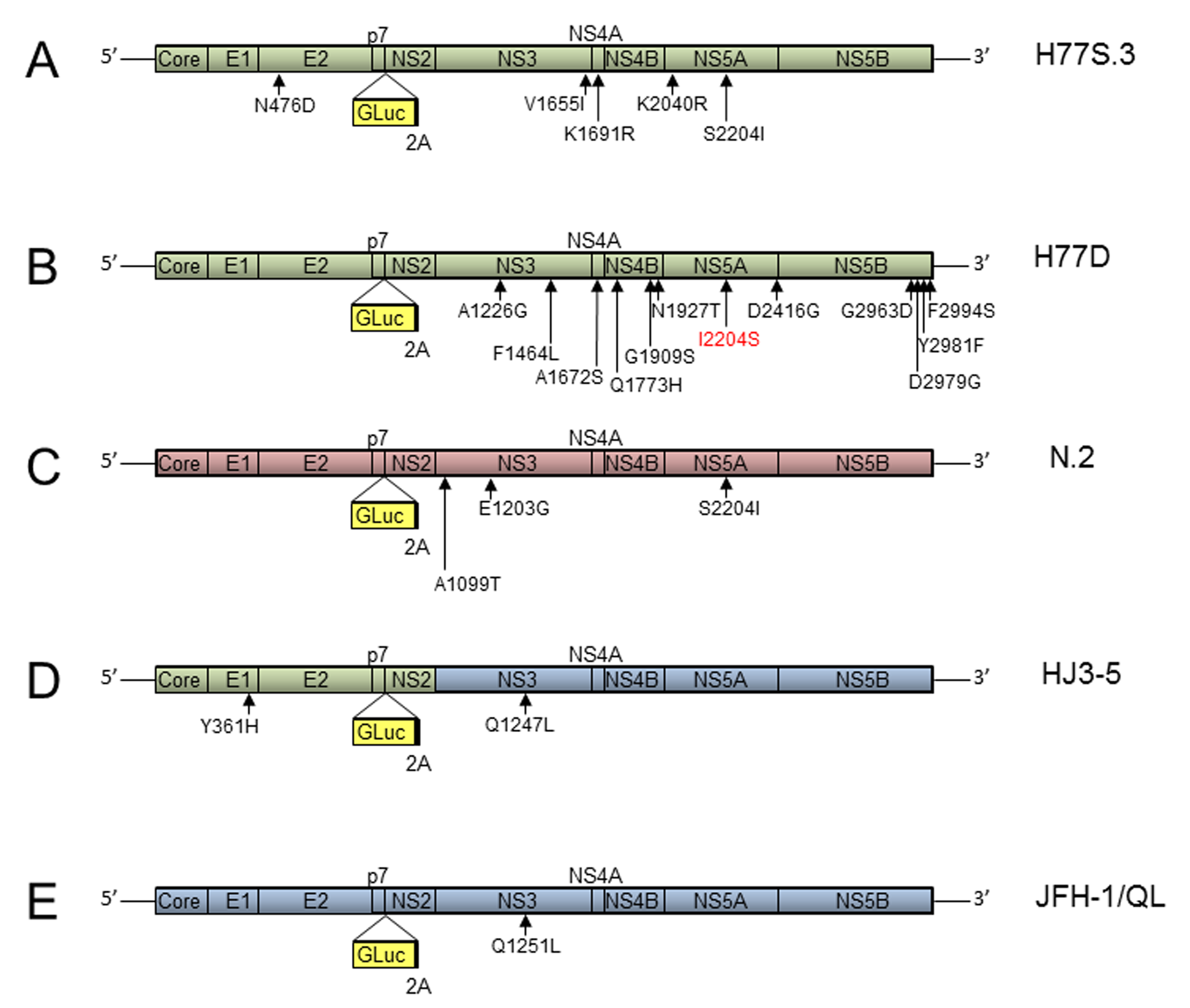

Supplement: S11 Fig — (A) H77S.3/GLuc2A, derived from gt 1a strain H77, Genbank Accession AF011751, and containing the indicated cell culture adaptive mutations. (B) H77D/GLuc2A, derived from H77S.3/GLuc2A. Amino acid positions that differ from H77S.3/GLuc2A are indicated. All are cell culture adaptive mutations except I2204S, shown red, which is mutated back to the original H77 sequence. (C) N.2/GLuc2A, derived from gt1b HCV-N strain, Genbank Accession AF139594, and containing the indicated cell culture adaptive mutations. (D) HJ3-5/GLuc2A, a chimeric gt1a/2a virus containing core to NS2 sequence from H77 and NS3 to NS5B sequence from JFH-1 with compensatory mutations in E1 and NS3. (E) JFH-1/QL/GLuc2A, derived from gt2a strain JFH-1, Genbank Accession AB047639, and containing the indicated cell culture adaptive mutation. (TIF) [file ppat.1006343.s011.tif]
