# Supplementary material for: NS5A inhibitors unmask differences in functional replicase complex half-life between different hepatitis C virus strains
Source: PLoS Pathog. 2017 Jun 8;13(6):e1006343. doi: 10.1371/journal.ppat.1006343 (PMC5464671; doi:10.1371/journal.ppat.1006343)
Supplement: S1 Text — (DOCX) [file ppat.1006343.s012.docx]

**Supporting Information for NS5A inhibitors unmask differences in functional replicase complex half-life between different hepatitis C virus strains**

**T. Benzine, R. Brandt, W. C. Lovell, D. Yamane, P. Neddermann, R. De Francesco, S. M. Lemon, A. S. Perelson, R. Ke and D. R. McGivern**

**Analytical approximations to the mathematical model**

To determine which parameter(s) drives the decrease in GLuc activity under treatment and thus assess the robustness of our parameter estimation, we derived analytical approximations to the mathematical model. Our analysis below focuses on the analytical solutions for the time evolution of the +RNAs in the cytosol (*T* in the model), because the decline in the level of GLuc activity is mainly driven by the decline of the +RNAs serving as translation templates in the cytosol.

We consider the scenario of a high drug concentration by assuming that the efficacy of a drug under consideration is 100%, i.e. $\varepsilon_{D}=1$. Under this assumption, the system becomes linear and the long-term exponential decline rate can be found by finding the largest eigenvalue of the matrix corresponding to the linear system of equations.

For elbasvir treatment, we take $\left( 1-\varepsilon_{ELB} \right)=0$ and set $\varepsilon_{C23}=\varepsilon_{SOF}=0$, and write the system of linear ordinary differential equations (ODEs) as:

$\left[ \begin{matrix} T(t) \\ R(t) \\ C(t) \\ A(t) \end{matrix} \right]\boldsymbol{'}=\boldsymbol{M}\left[ \begin{matrix} T(t) \\ R(t) \\ C(t) \\ A(t) \end{matrix} \right]$, where $\boldsymbol{M=}\left[ \begin{matrix} -(\sigma+\delta\boldsymbol{)} & \theta& 0 & \eta\\ \sigma& -(\theta+k+\delta) & \alpha& 0 \\ 0 & 0 & -\mu& 0 \\ 0 & k & 0 & -(\delta+\eta) \end{matrix} \right]$

The eigenvalues of *M* are:

$$\lambda_{M1}=-\mu$$

$$\lambda_{M2}=-\delta$$

$\lambda_{M3}=-\frac{1}{2}\left[ 2\delta+k+\sigma+\theta+\eta-\sqrt{{(k+\sigma+\theta+\eta)}^{2}-4k\sigma-4k\eta-4\sigma\eta-4\theta\eta} \right]$

$$\lambda_{M4}=-\frac{1}{2}\left[ 2\delta+k+\sigma+\theta+\eta+\sqrt{{(k+\sigma+\theta+\eta)}^{2}-4k\sigma-4k\eta-4\sigma\eta-4\theta\eta} \right]$$

It can be shown that $\lambda_{M2}=-\delta>\lambda_{M3}>\lambda_{M4}$. Since the estimated value of $\mu$ is less than the value of $\delta$, we have $\lambda_{M1}>\lambda_{M2}>\lambda_{M3}>\lambda_{M4}$. Thus, the rate of long-term exponential decrease of the GLuc protein under elbasvir treatment is determined by the largest eigenvalue, $-\mu$.

For Compound 23 treatment, we let $\left( 1-\varepsilon_{C23} \right)=0$ and $\varepsilon_{ELB}=\varepsilon_{SOF}=0$. Then, the system can be written as the matrix equation:

$\left[ \begin{matrix} T(t) \\ R(t) \\ C(t) \\ A(t) \end{matrix} \right]\boldsymbol{'}=\boldsymbol{N}\left[ \begin{matrix} T(t) \\ R(t) \\ C(t) \\ A(t) \end{matrix} \right]$, where $\boldsymbol{N=}\left[ \begin{matrix} -(\sigma+\delta\boldsymbol{)} & \theta& 0 & \eta\\ \sigma& -(\theta+k+\delta) & \alpha& 0 \\ 0 & 0 & -\mu& 0 \\ 0 & k & 0 & -(\delta+\rho+\eta) \end{matrix} \right]$

One eigenvalue of *N* is:

$$\lambda_{N1}=-\mu$$

The other three eigenvalues of *N* are solvable, but cannot be easily expressed. We denote these other three eigenvalues as $\lambda_{N2}, \lambda_{N3}$ and $\lambda_{N4}$.

For sofosbuvir, we let $\left( 1-\varepsilon_{SOF} \right)=0$ and $\varepsilon_{ELB}=\varepsilon_{C23}=0$. Then, the equation for *C*, $\frac{dC}{dt}=-\mu C$, decouples from the other equations, and the dynamics of *C* does not impact the dynamics of +RNAs and thus the GLuc activity. The remaining system can be written as the matrix equation:

$\left[ \begin{matrix} T(t) \\ R(t) \\ A(t) \end{matrix} \right]^{\boldsymbol{'}}=\boldsymbol{W}\left[ \begin{matrix} T(t) \\ R(t) \\ A(t) \end{matrix} \right]$, where $\boldsymbol{W=}\left[ \begin{matrix} -(\sigma+\delta\boldsymbol{)} & \theta& \eta\\ \sigma& -(\theta+k+\delta) & 0 \\ 0 & k & -(\delta+\rho+\eta) \end{matrix} \right]$

Interestingly, the resulting three eigenvalues of *W* are identical to $\lambda_{N2}, \lambda_{N3}$ and $\lambda_{N4}$.

The analysis for Compound 23 and sofosbuvir treatments above suggests that if any of the eigenvalues $\lambda_{N2}, \lambda_{N3}$ and $\lambda_{N4}$ is larger than $\lambda_{N1}$(i.e. $-\mu$), the long-term decrease rates under effective Compound 23 and sofosbuvir treatments would be identical. However, our data clearly show that the level of GLuc protein declines at a slower rate under Compound 23 treatment than under sofosbuvir treatment, which suggests that $\lambda_{N1}$ is greater than $\lambda_{N2}, \lambda_{N3}$ and $\lambda_{N4}$, and the decline rate of Gluc protein under Compound 23 treatment is driven by the rate of the RC becoming nonfunctional and degraded, i.e. the parameter $\mu$.

Altogether, this analysis above shows that the value of $\mu$, i.e. the rate that the RC becomes nonfunctional and degraded can be reliably estimated by fitting the model to the elbasvir and Compound 23 treatment data. In addition, fitting the model to the time course data collected from all three treatments, we will be able to estimate parameter values of other processes, although the estimated parameter values may depend on fixed parameter values in complicated ways.
